# Morphologic, phenotypic, and transcriptomic characterization of classically and alternatively activated canine blood-derived macrophages *in vitro*

**DOI:** 10.1371/journal.pone.0183572

**Published:** 2017-08-17

**Authors:** Franziska Heinrich, Annika Lehmbecker, Barbara B. Raddatz, Kristel Kegler, Andrea Tipold, Veronika M. Stein, Arno Kalkuhl, Ulrich Deschl, Wolfgang Baumgärtner, Reiner Ulrich, Ingo Spitzbarth

**Affiliations:** 1 Department of Pathology, University of Veterinary Medicine Hannover Foundation, Bünteweg 17, Hannover, Germany; 2 Center for Systems Neuroscience, Bünteweg 2, Hannover, Germany; 3 Department of Small Animal Medicine and Surgery, University of Veterinary Medicine Hannover Foundation, Bünteweg 2, Hannover, Germany; 4 Department of Clinical Veterinary Sciences, Vetsuisse Faculty, University of Bern, Laenggassstrasse 128, Bern, Switzerland; 5 Boehringer Ingelheim Pharma GmbH & Co.KG, Department of Non-clinical Drug Safety, Birkendorfer Str. 65, Biberach, Germany; 6 Friedrich-Loeffler-Institute, Department of Experimental Animal Facilities and Biorisk Management, Südufer 10, Greifswald, Germany; University of Michigan Health System, UNITED STATES

## Abstract

Macrophages are a heterogeneous cell population playing a pivotal role in tissue homeostasis and inflammation, and their phenotype strongly depends on the micromilieu. Despite its increasing importance as a translational animal model for human diseases, there is a considerable gap of knowledge with respect to macrophage polarization in dogs. The present study comprehensively investigated the morphologic, phenotypic, and transcriptomic characteristics of unstimulated (M0), M1- (GM-CSF, LPS, IFNγ-stimulated) and M2- (M-CSF, IL-4-stimulated)-polarized canine blood-derived macrophages *in vitro*. Scanning electron microscopy revealed distinct morphologies of polarized macrophages with formation of multinucleated cells in M2-macrophages, while immunofluorescence employing literature-based prototype-antibodies against CD16, CD32, iNOS, MHC class II (M1-markers), CD163, CD206, and arginase-1 (M2-markers) demonstrated that only CD206 was able to discriminate M2-macrophages from both other phenotypes, highlighting this molecule as a promising marker for canine M2-macrophages. Global microarray analysis revealed profound changes in the transcriptome of polarized canine macrophages. Functional analysis pointed out that M1-polarization was associated with biological processes such as “respiratory burst”, whereas M2-polarization was associated with processes such as “mitosis”. Literature-based marker gene selection revealed only minor overlaps in the gene sets of the dog compared to prototype markers of murine and human macrophages. Biomarker selection using supervised clustering suggested *latexin (LXN)* and *membrane-spanning 4-domains*, *subfamily A*, *member 2 (MS4A2)* to be the most powerful predicting biomarkers for canine M1- and M2-macrophages, respectively. Immunofluorescence for both markers demonstrated expression of both proteins by macrophages *in vitro* but failed to reveal differences between canine M1 and M2-macrophages. The present study provides a solid basis for future studies upon the role of macrophage polarization in spontaneous diseases of the dog, a species that has emerging importance for translational research.

## Introduction

Circulating peripheral blood mononuclear cells (PBMCs) play an important role during both the steady state and inflammation. Monocytes, which originate from hematopoietic stem cells, are capable of migrating from the blood into distinct tissues and differentiate into macrophages in order to replenish specific tissue-specific macrophage populations [1]. Functional diversity and plasticity are hallmarks of macrophages [2, 3]. Together they represent a heterogeneous cell population of the mononuclear phagocyte system playing a pivotal role in tissue homeostasis, inflammation, host defense, and tissue repair [4, 5]. Depending on the micromilieu, two extremes of macrophage phenotypes have been described following external or endogenous stimulation: “classically” activated M1-macrophages and “alternatively” activated M2-macrophages [6, 7]. Classically activated M1-macrophages develop after exposure to pro-inflammatory stimuli such as interferon ɣ (IFNɣ), lipopolysaccharide (LPS), or tumor necrosis factor (TNF). Subsequent to such stimulation, M1-macrophages release pro-inflammatory cytokines, reactive oxygen species (ROS), and nitric oxide (NO) [8]. Hence, on the functional level, M1-macrophages are characterized by an increased microbicidal, tumoricidal, and antigen presenting capacity [2, 4, 9]. In contrast, M2-macrophages become activated in the presence of interleukin (IL)-4, IL-10, IL-13, glucocorticoids, and transforming growth factor β (TGFβ) leading to enhanced secretion of anti-inflammatory cytokines. Accordingly, M2-macrophages are functionally associated with hypersensitivity, parasite clearance, inflammatory dampening, tissue remodeling, angiogenesis, immunoregulation, and tumor promotion [2, 9, 10]. However, it should be taken into consideration that the M1-/M2–paradigm is a simplified classification, representing only two extremes of phenotypes which do not fully mirror the complexity of the dynamic biological processes behind cell polarization [7]. Hence, gene expression profiling has been applied as a sophisticated technique to detect the underlying molecular mechanisms following macrophage activation in murine and human cells [11–14]. In fact, macrophage activation by any agonist involves a massive change in gene expression during the transition from one steady state to another [5]. Notably, current comparative studies strongly support the observation of marked interspecies differences and variability between mice and humans indicating that about 50% of polarization specific markers are selectively expressed in only one of both species [13, 14].

Currently, spontaneous diseases in dogs play an established and increasing role as suitable animal models for human disorders including for instance demyelinating central nervous system (CNS) diseases, measles, cancer, and spinal cord injury (SCI) [15–21]. Conclusively, dogs represent a promising so called large animal model in the development of novel therapeutic approaches for naturally occurring diseases. However, despite the essential role of dogs as a translational animal model, there is a considerable lack of knowledge of the role of macrophage polarization in this species, and morphologic, phenotypic, and transcriptomic properties of polarized canine macrophages are enigmatic so far. However, a detailed knowledge of these basic principles doubtlessly represents a prerequisite for envisaged pharmacotherapeutic and cell transplantation studies.

Therefore, this *in vitro* study aimed to (i) characterize morphological differences of polarized canine macrophages, (ii) to test the capability of established murine and human prototypical markers to differentiate canine M1- and M2-macrophages using immunofluorescence, and (iii) to unravel differences in the transcriptome of canine polarized macrophages using microarray technique in order to establish unique gene signatures, which differentiate these polarization states.

The presented results provide a highly needed basis for future research upon canine spontaneous diseases such as SCI and distemper leukoencephalitis with a special emphasis upon the so far enigmatic role of macrophage polarization in these diseases.

## Materials and methods

### Blood cell isolation

Blood samples were collected from a total number of 12 healthy Beagle dogs, which were kept in regulatory approved animal housing facilities of the Departments of Small Animal Medicine and Surgery and the Institute for Parasitology, University of Veterinary Medicine, Hannover, Germany. Blood collection was done by professional veterinarians from non-anesthetized dogs following the regulations of the German Animal Welfare Law and with permission provided by the Niedersächsisches Landesamt für Verbraucherschutz und Lebensmittelsicherheit, Oldenburg, Germany (permission number: AZ33.9-42502-05-13A303). Following a clinical examination of each animal, which included auscultation of the lung and heart, and rectal measurement of the body temperature, the puncture spot was disinfected. A volume of 20 ml blood was collected from the cephalic vein of each dog. Following blood collection, local manual pressure was used to avoid bleeding. No animals were euthanized for the present study.

Peripheral blood mononuclear cells (PBMCs) were isolated via density gradient centrifugation using a routine protocol as described previously [22]. Briefly, blood samples were diluted 1:3 in phosphate buffered saline (PBS) containing 1% penicillin/streptomycin (Biochrom GmbH, Berlin, Germany) and added onto a gradient with equal volumes of histopaque 1.077 g/ml and 1.119 g/ml (Sigma Aldrich, Taufkirchen, Germany). After 30 minutes of centrifugation, cells were harvested using a Pasteur pipette, washed with PBS and 1% penicillin/streptomycin followed by depletion of erythrocyte contamination by hypotone lysis with distilled water. Subsequently, cells were resuspended in Roswell Park Memorial Institute (RPMI)-medium 1640 (Biochrom GmbH, Berlin, Germany) containing 10% fetal calf serum (PAA, Cölbe, Germany) and 1% penicillin/streptomycin and seeded onto 96½-well plates (ThermoScientific, Waltham, MA, USA) at a density of 0.3 x 10^6 cells/well. After 24 hours of cultivation under standard conditions (5% CO_2_, 37°C), medium was completely removed and cells were washed twice with PBS and 1% penicillin/streptomycin. Cells attached to the base of wells with strong plastic adherence were referred to as monocytes [23–26].

### Cell culture and polarization

To culture canine macrophages *in vitro* from blood-derived monocytes, a protocol according to Durafourt et al. (2012) [27] was used with slight modifications. Briefly, canine monocytes were cultured over 7 days towards M1-/M2-macrophages by stimulation with hematopoietic growth factors over 5 days followed by activation over 2 days with distinct cytokines, purchased from companies that guarantee an endotoxin level below 0.1 EU/μg of protein. In particular, to obtain M1-macrophages, monocytes were treated with 5 ng/ml canine recombinant granulocyte macrophage colony-stimulating factor (GM-CSF; R&D Systems, Minneapolis, MN, USA), 100 ng/ml LPS (Sigma Aldrich, Taufkirchen, Germany), and 20 ng/ml recombinant canine IFNɣ (Kingfisher Biotech Inc., Saint Paul, Minnesota, USA). M2-macrophages were developed by treatment with 25 ng/ml human macrophage colony-stimulating factor (M-CSF, PeproTech Inc., Rocky Hill, NJ, USA) and 20 ng/ml canine recombinant IL-4 (R&D Systems, Minneapolis, MN, USA). Serving as a control, unstimulated monocytes were cultured under the same conditions with medium change every second day (M0-macrophages). After 7 days of cultivation, morphology, phenotype, and transcriptomic changes of M0-, M1-, and M2-macrophages were assessed. Pictures were taken with an inverted fluorescence microscope (Olympus IX-70, Olympus, Optical Co GmbH, Hamburg, Germany).

The overall number of cells for each polarization was counted on at least 4 microscopic pictures (200x magnification) of three individual dogs on day 7 in culture and the mean numbers of cells per 200x field were compared using one-factorial ANOVA and post-hoc t tests applying SPSS version 21 for windows (IBM Inc., Chicago, USA).

For quantification of cellular morphology, microphotographs were taken at 200x magnification and the number of cells with a distinct morphologic appearance out of at least 100 randomly selected cells was counted for each polarization (M0, M1, M2). Here, 4 distinct morphologic types were defined. In particular, cells with a cellular diameter ≤ 10 μm and absent cytoplasmic projections on the cellular surface were defined as small/roundish cells (morphology 1); cells with a cellular diameter ≥ 10 μm and numerous cytoplasmic processes were defined as amoeboid (morphology 2); cells obtaining an elongated bipolar morphology were classified as spindeloid (morphology 3); and cells possessing a large, round, and flattened morphology with a cellular diameter ≥ 30 μm and > 1 nuclei were classified as multinucleated giant cells (MNGs; morphology 4).

The percentage of each of the four different morphologies was calculated in M0-, M1-, and M2-macrophages from 3 animals. A mixed ANOVA with post-hoc alpha adjustment (Tukey-Kramer) and significance level at p≤0.05 was performed using the statistical software programme SPSS version 21 for windows (IBM Inc., Chicago, USA) in order to compare the percentages of each of the morphologies between M0-, M1-, and M2-macrophages. Graphical compilation was done with GraphPad Prism 5.0 (GraphPad Software Inc., La Jolla, USA).

### Scanning electron microscopy

M0-, M1-, and M2-macrophages were prepared for representative ultrastructural characterization using scanning electron microscopy as described previously [28]. Briefly, cells were fixed in 2.5% glutaraldehyde/cacodylate buffer, post-fixed in 1% osmium tetroxide, and dehydrated in a graded series of alcohol. Subsequently, cells were dried under a Critical Point Dryer (E3000, Polaron, London, UK), and sputter-coated with gold, and examined under a digital scanning electron microscope (DSM 940, Zeiss, Oberkochen, Germany). At least 50 cells were photographed for each morphology and evaluated with regard to ultrastructural morphology.

### Immunocytochemistry

Immunolabelling of canine M0-, M1-, and M2-macrophages from 5 individuals was performed using a routine protocol described previously [29, 30]. A panel of different literature-based prototype marker antibodies, reported to distinguish between M1- and M2-macrophages was applied (Table 1; [4, 31]). Briefly, cells were fixed with paraformaldehyde (PFA, 4%) for 20 minutes at room temperature (RT) and permeabilized with Triton X (0.25%) diluted in PBS (PBST). Non-specific binding was blocked by treatment of cells with bovine serum albumin (BSA, 3%; Sigma Aldrich, Taufkirchen, Germany) and normal goat serum (5%) diluted in PBST for 15 minutes at RT, except for cells intended to be stained with antibodies directed against Fc-receptors, *i*.*e*. CD16 and CD32. Subsequently, primary antibodies (Table 1) were added and incubated for 2 hours at RT. All unconjugated primary antibodies were labeled with secondary goat-anti-mouse antibodies coupled to cyanine 3 (Cy3), goat-anti-rat antibodies coupled to Cy2, and goat-anti-rabbit antibodies coupled to Cy3 (all received from Jackson ImmunoResearch Laboratories, Dianova, Hamburg, Germany; dilution 1:200 in PBS), respectively, and incubated for 2 hours at RT. Following appropriate washing steps with PBS and distilled water, nuclei were counterstained with bisbenzimide (H33258, 0.01% in distilled water) for 5 minutes at RT. Species-specific immunoglobulins from mouse, rat, goat, and rabbit diluted according to the immunoglobulin concentration of the primary antibodies served as appropriate negative controls. M0-, M1-, and M2-macrophages were investigated using an inverted fluorescence microscope (Olympus IX-70, Olympus, Optical Co GmbH, Hamburg, Germany). For quantification of immunopositivity, microphotographs were taken at 200x magnification and at least 100 randomly selected cells were counted for each treatment (M0, M1, M2) with respect to the number of immunopositive cells and the total number of cells. Statistical comparison of the percentage of immunopositive cells between M0-, M1-, and M2-macrophages was done using a Kruskal-Wallis-Test and pairwise Mann-Whitney-U-Tests with significance level at p≤0.05 employing the statistical software programme SPSS version 21 for windows, and box plots were depicted using GraphPad Prism 5.0 (GraphPad Software Inc., La Jolla, USA).

**Table 1:** List of antibodies used for immunofluorescence.

Polarity	Antigen	Clone	Clonality	Source	Dilution
M1	CD16	LNK16	Monoclonal mouse	Abcam‡	1:20
	CD32	AT10	Monoclonal mouse	Abcam‡	1:10
	MHC class II	Dog 26	Monoclonal rat	Helmholtz Zentrum†	1:10
	iNOS	n.a.	Polyclonal rabbit	Merck Milipore᛭	1:50
	LXN	n.a.	Polyclonal goat	Biologo *	1:10
M2	MS4A2	n.a.	Polyclonal rabbit	Biologo *	1:10
	CD163	AM-3K	Monoclonal mouse	TransGenic Inc. ‖	1:20
	CD206	3.29B1.10	Monoclonal mouse	BeckmanCoulter Inc.¶	1:20
	Arginase-1	n.a.	Polyclonal rabbit	Sigma Aldrich§	1:125

CD, cluster of differentiation; MHC, major histocompatibility complex; iNOS, inducible nitric oxide synthase, LXN, latexin; n.a. = not applicable

‡Cambridge, UK

†kindly provided by Dr. E Kremmer, Institute of Molecular Immunology, Helmholtz Zentrum, München, German Research Center for Environmental Health (GmbH), Munich, Germany

᛭Darmstadt, Germany

‖Kobe, Japan

¶Krefeld, Germany

§ Taufkirchen, Germany

* Kronshagen, Germany

Based on the results of the transcriptome investigations (see below), two additional antibodies targeting LXN and MS4A2 (Table 1) were applied on isolated and polarized cells of 3 dogs. Immunofluorescence was performed analogously to the above mentioned methods. For these antibodies, blocking was done with normal horse serum. Donkey-anti-goat and donkey-anti-rabbit antibodies were used as secondary antibodies. Due to the lower n (3 dogs), statistical comparison of the percentage of immunopositive cells between M0-, M1-, and M2-macrophages was done using parametrical tests (one-factorial ANOVA and pairwise t tests) for these both antibodies.

### RNA isolation, microarray hybridization, and low level analysis

For RNA-isolation a separate and analogous *in vitro* experiment was performed using blood-derived cells from 6 healthy Beagles. Total RNA was isolated from 6 biological replicates of M0-, M1- and M2- macrophages using the RNeasy Mini Kit according to the manufacturer’s instructions (Qiagen, Hilden, Germany). Quality and integrity of isolated RNA were controlled using the Agilent 6000 RNA Nano Kit and an Agilent Bioanalyzer 2100 (Agilent, Böblingen, Germany). 200 ng RNA of each sample was amplified and biotin-labeled employing the Ovation RNA Amplification Kit V2 and the Encore Biotin Module (NuGen, San Carlos, USA), and hybridized to GeneChip Canine Genome 2.0 arrays (Affymetrix, Santa Clara, USA) in a rotating oven (45°C, 16 hours). Subsequently, arrays were washed and stained with R-phycoerythrin-streptavidin employing Affymetrix GeneChip Fluidics Station 450 (Affymetrix, Santa Clara, USA). For scanning, an Affymetrix GeneChip Scanner 3000 (Affymetrix, Santa Clara, USA) was employed for signal detection. Background adjustment, quantile normalization, and probe-set summarization were performed using the Gene Chip Robust Multichip Average (GC-RMA) algorithm (Bioconductor *gcrma* for R package, Version 2.3) as previously described [32]. Raw and processed data sets of the present study are deposited and publically available in the ArrayExpress database (accession number: E-MTAB-5458; http://www.ebi.ac.uk/arrayexpress).

### Differentially expressed probe sets

Differentially expressed probe sets (DEPs) were detected using the Linear Models for Microarray Data (LIMMA) algorithm, implemented in Babelomics 4.3 (http://babelomics.bioinfo.cipf.es; [33]), with a maximal false discovery rate (FDR) of 5% (q≤0.05) according to Benjamini and Hochberg, followed by *post-hoc* pairwise comparison of the expression levels of M0-, M1-, and M2-macrophages [32, 34]. The fold change (FC) was calculated as the ratio of the inverse-transformed arithmetic means of the log_2_-transformed expression values. Down-regulations are shown as negative reciprocal values [34, 35]. Probe sets were annotated with canine gene symbols and gene names according to the Affymetrix annotation file (release 35; 06. October 2014). A statistical significance filter (LIMMA q≤0.05) and a fold change filter (FC≥2.0 or ≤2.0) were employed to identify differentially expressed probe sets (DEPs) [32]. Differentially expressed genes (DEGs) were defined as probe sets with a unique canine gene symbol annotation [36].

### Functional annotation and hierarchical clustering analysis

Functional enrichment analysis of the DEPs for overexpressed Gene Ontology (GO) terms of the biological process category and the Kyoto encyclopedia of genes and genomes (KEGG) was performed using Web-based Gene Set Analysis Toolkit (WebGestalt; http://bioinfo.vanderbilt.edu/webgestalt/; [37–39]). In order to detect the genes in the pairwise comparison of M0-, M1-, and M2-macrophages, whose expression was most severely affected, genes with a FC≥50.0 or ≤-50.0 were selected and grouped into biological categories [36].

Moreover, unsupervised hierarchical clustering analysis of the DEPs was performed on log_2_-transformed data using TM4 MultiExperimentViewer (MeV) with default settings (Euclidean distance and complete linkage) [40]. The list of DEPs for each of the resulting clusters was functionally analyzed for significant enrichment applying WebGestalt as described above.

### Correlation-based marker gene selection

Based on the transcriptional profile, a subset of probe sets for polarization prediction was selected using Prophet [41], provided by Babelomics 4.3 [33, 42]. Correlation-based feature selection was used to pre-select the informative subset of probe sets and K-nearest-neighbors (KNN) algorithm was used for class prediction with leave-one out error validation [42]. The informative subset of 369 probe sets derived from Prophet was further analyzed for the polarization prediction strength of each individual probe set in three independent “M0 *versus* M1 and M2”, “M1 *versus* M0 and M2”, and “M2 *versus* M0 and M1” tests using Signature evaluation tool (SET; [42, 43]). The log_2_-transformed expression values of three biomarkers, suggested by Prophet, were additionally statistically compared between M0-, M1-, and M2-macrophages, employing Kruskal-Wallis-Test and subsequent pairwise Mann-Whitney-U-Tests with significance value p≤0.05.

### Comparative evaluation of canine M1-/M2-genes with established literature-based human and murine orthologous genes

All unique gene symbols, which were up-regulated (FC≥2; q≤0.05) in the comparison of M1 *vs*. M0 and simultaneously up-regulated in the comparison of M1 *vs*. M2 were defined as M1-exclusive-genes (n = 404). Likewise, all unique gene symbols, which were up-regulated (FC≥ 2; q≤0.05) in the comparison of M2 *vs*. M0 and simultaneously up-regulated in the comparison of M2 *vs*. M1 were defined as M2-exclusive-genes (n = 700). The gene sets of these exclusive M1- or M2-associated canine genes were compared with a list of established human and murine genes specifically associated with M1- or M2-macrophages. This list was generated based on peer-reviewed publications [4, 27, 31, 44] as previously described [8, 45]. This list was translated into canine orthologous gene symbols by employing MADgene (http://cardioserve.nantes.inserm.fr/madtools/madgene/; [46]) and missing orthologous canine official gene symbols were manually added with the help of the web-based “information hyperlinked over proteins” (ihop; http://www.ihop-net.org/UniPub/iHOP/; [47]) as described previously [36]. Summarized, the literature-based gene list included a total number of 65 orthologous canine genes for M1-macrophages and 58 orthologous canine genes for M2-macrophages [36]. Venn diagrams (http://bioinfogp.cnb.csic.es/tools/venny/index.html) were used to reveal the intersections between established literature-based genes and the gene sets identified by the present analysis.

## Results

### Morphological characterization of polarized canine macrophages

On day 7 in culture, the overall number of cells differed between the three polarities (p = 0.02; S1 Fig). The number of cells was highest in M2-polarized cells (mean = 135 cells per field), while M0-macrophages were lowest in number (mean = 30 cells per field). M1-macrophages had a mean number of 52 cells per field. T tests revealed that the number of M2-macrophages was significantly higher than M0-macrophages (p = 0.02), while comparisons between M0- and M1-macrophages and M1-and M2-macrophages failed to reach the level of significance (p = 0.30 and p = 0.06, respectively).

**Fig 1:**
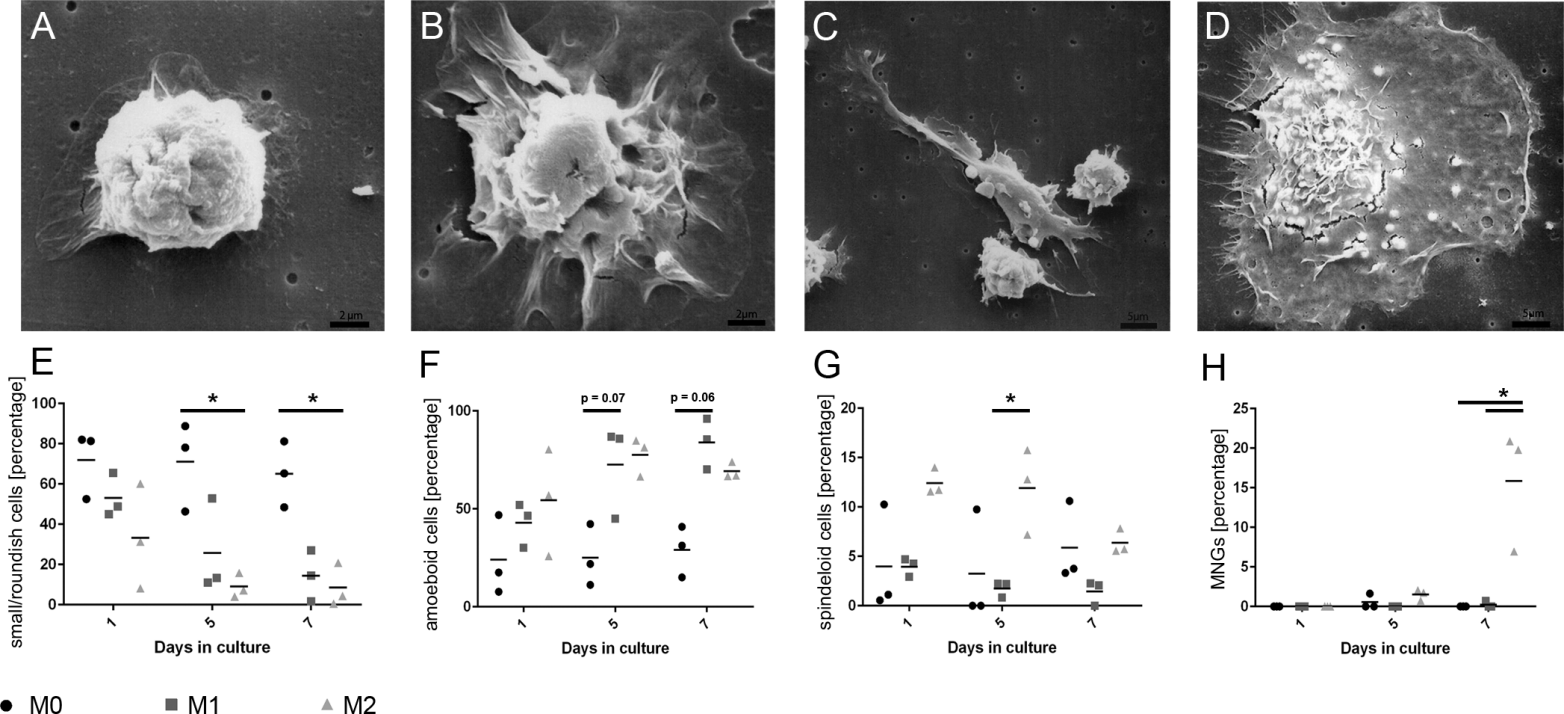
Polarization-dependent morphological differences in canine M0-, M1-, and M2-macrophage cultures. **A)** In scanning electron microscopy, unstimulated macrophages (M0; day 7) obtain a small and roundish morphology, lacking cytoplasmic extensions. **B)** M1-treated macrophages (day 7) are characterized by an enlarged amoeboid cell shape with roundish cell bodies and numerous delicate cytoplasmic extensions on the cellular surface. **C; D)** M2-treated macrophage cultures (day 7) demonstrate a marked heterogeneity with two dominating cell types. Large “spindeloid” macrophages with an elongated cell body and cytoplasmic extensions on the apical ends of the cell bodies **(C)**. Second, in M2-cultures, numerous multinucleated giant cells (MNGs) with abundant cytoplasmic projections on the cellular surface are present **(D)**. **E-H)** Dot plot diagrams depicting the morphological changes of macrophage cultures (n = 3) following stimulation (M0, M1, M2) over the time course as calculated with a mixed ANOVA with post-hoc alpha adjustment (Tukey-Kramer) and significance level at p≤0.05 (asterisks). **E)** Small/roundish macrophages dominate in untreated M0 cell cultures and their relative percentage is significantly higher in M0 when compared to M2 at days 5 and 7. **F)** Amoeboid macrophages display a statistical trend of predominance in M1 compared to M0 at day 5 and 7. **G)** The relative percentage of spindeloid macrophages is significantly increased in M2 at day 5 compared to M1. **H)** MNGs are almost exclusively observed in M2-macrophages at the end of the culturing period and their percentage is significantly higher at day 7 in M2 compared to both M0 and M1.

Morphologically, striking differences between M0-, M1-, and M2-polarized cells were observed at day 7 in culture by scanning electron microscopy and phase contrast microscopy (Fig 1). Canine M0-macrophages predominantly appeared as small and roundish cells with an average size of 8 μm in diameter and no or little cytoplasmic extensions that measured up to 1 μm in length (Fig 1). However, a noteworthy proportion of M0-macrophages (about 25%) also obtained an amoeboid morphology, which however was predominantly observed in M1 macrophages (Fig 1). The majority of M1-macrophages was amoeboid and had a mean size of 15 μm and numerous fibrillary cytoplasmic processes on the cellular surface that had a length of up to 5 μm (Fig 1). M2-macrophages appeared as a heterogeneous cell population with a mixture of 4 different morphologies. Besides roundish and amoeboid macrophages, large bipolar spindeloid macrophages were present measuring up to 35 μm that were characterized by an elongated cell body with cytoplasmic extensions with an average length of 7 μm at both poles (Fig 1). Moreover, large MNGs appeared that had a mean size of 40 μm with an extensive cytoplasm and numerous evenly distributed processes that measured up to 7 μm (Fig 1). Allover, the mean percentage of small/roundish macrophages (morphology 1) was significantly higher in untreated cultures (M0) compared to polarity 2 at days 5 and 7 (p≤0.05; Fig 1E). For amoeboid macrophages (morphology 2), there was a trend towards a higher mean percentage in polarity 1 compared to polarity 0 at day 5 (p = 0.07) and day 7 (p = 0.06; Fig 1F). The mean percentage of spindeloid macrophages (morphology 3) was significantly higher in polarity 2 at day 5 compared to polarity 1 at day 5 (p≤0.05; Fig 1G). MNGs (morphology 4) were exclusively present in polarity 2, as compared to the polarities 0 and 1 at day 7 (p≤0.05; Fig 1H).

### Phenotypical characterization of polarized canine macrophages

**Fig 2:**
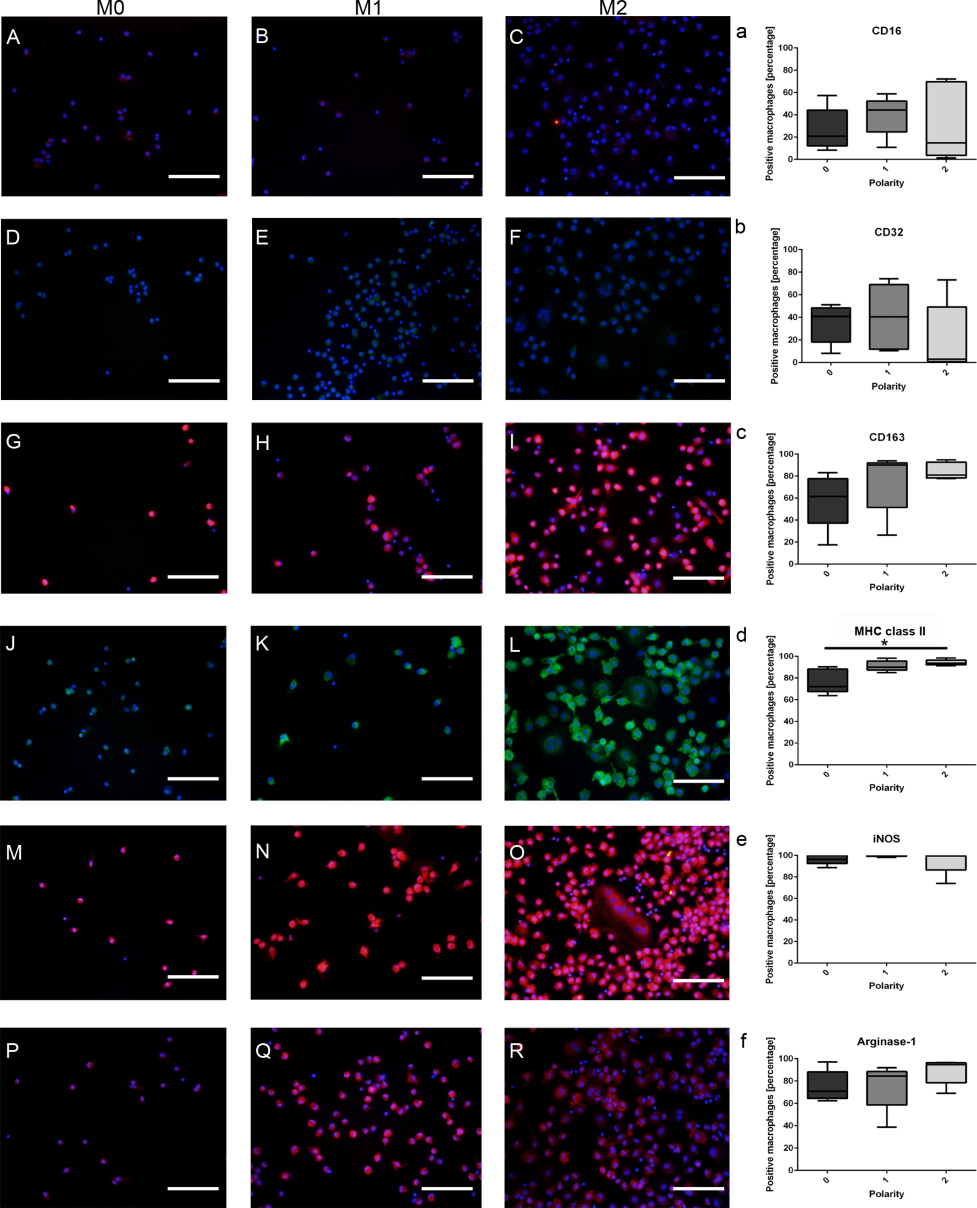
Immunofluorescence staining of *in vitro* cultured canine M0-, M1-, and M2-macrophages labeled with prototypic literature-based antibodies for the M1- (CD16, CD32, MHC class II, and iNOS) and M2-phenotype (CD163, CD206, and arginase 1), respectively. **A-C)** Low to moderate membranous staining of M0-, M1-, and M2-macrophages for CD16. **D-F)** Likewise, CD32 shows a low to moderate staining in all three treatment conditions. **G-I)** M0-, M1-, and M2-macrophages demonstrate a moderate to high membranous staining with CD163. **J-L)** Intense membranous staining of M0-, M1-, and M2-macrophages with an anti-MHC class II antibody. **M-O)** Strong intracytoplasmic labeling of macrophages in all treatments (polarity 0, 1, 2) for inducible nitric oxide synthase (iNOS). **P-R)** High intracytoplasmic expression of arginase 1 in small/roundish, amoeboid, and spindeloid macrophages as well as in multinucleated giant cells (MNGs). **a-f)** Statistical evaluation of the mean expression percentages of prototypic M1-/M2-markers evaluated in 5 dogs and related to the polarization state of the macrophages (polarity 0, 1, 2). Note that, except for MHC class II, none of the remaining tested antigens (CD16, CD32, iNOS, CD163, and arginase-1) were differently expressed between canine M0-, M1-, and M2-macrophages (* = p≤0.05; Kruskal-Wallis-Test with pairwise Mann-Whitney-U-Tests). Scale bars = 100 μm. Nuclear counterstaining with bisbenzimide.

For the phenotypical characterization of canine macrophages, the percentage of immunopositive cells for selected literature-based M1-/M2-antigens was evaluated in 5 animals and related to the polarization of cells (polarity 0, 1, 2). Interestingly, except for MHC class II and CD206, none of the remaining tested antigens (CD16, CD32, iNOS, CD163, and arginase-1) were differently expressed between canine M0-, M1-, and M2-macrophages (p-values ranging from 0.101 to 0.691; Kruskal-Wallis-Test; Fig 2).

The mean percentage of immunopositive cells expressing MHC class II was significantly higher in M2-macrophages (mean positive cells: 93.64%) when compared to untreated M0-cells (mean positive cells: 72.22%; p = 0.008). However, there was no statistical difference in the percentage of positive cells for MHC class II, when M1-macophages (mean positive cells: 89.92%) were compared to M2-macrophages and M0-macrophages, respectively (Fig 2).

**Fig 3:**
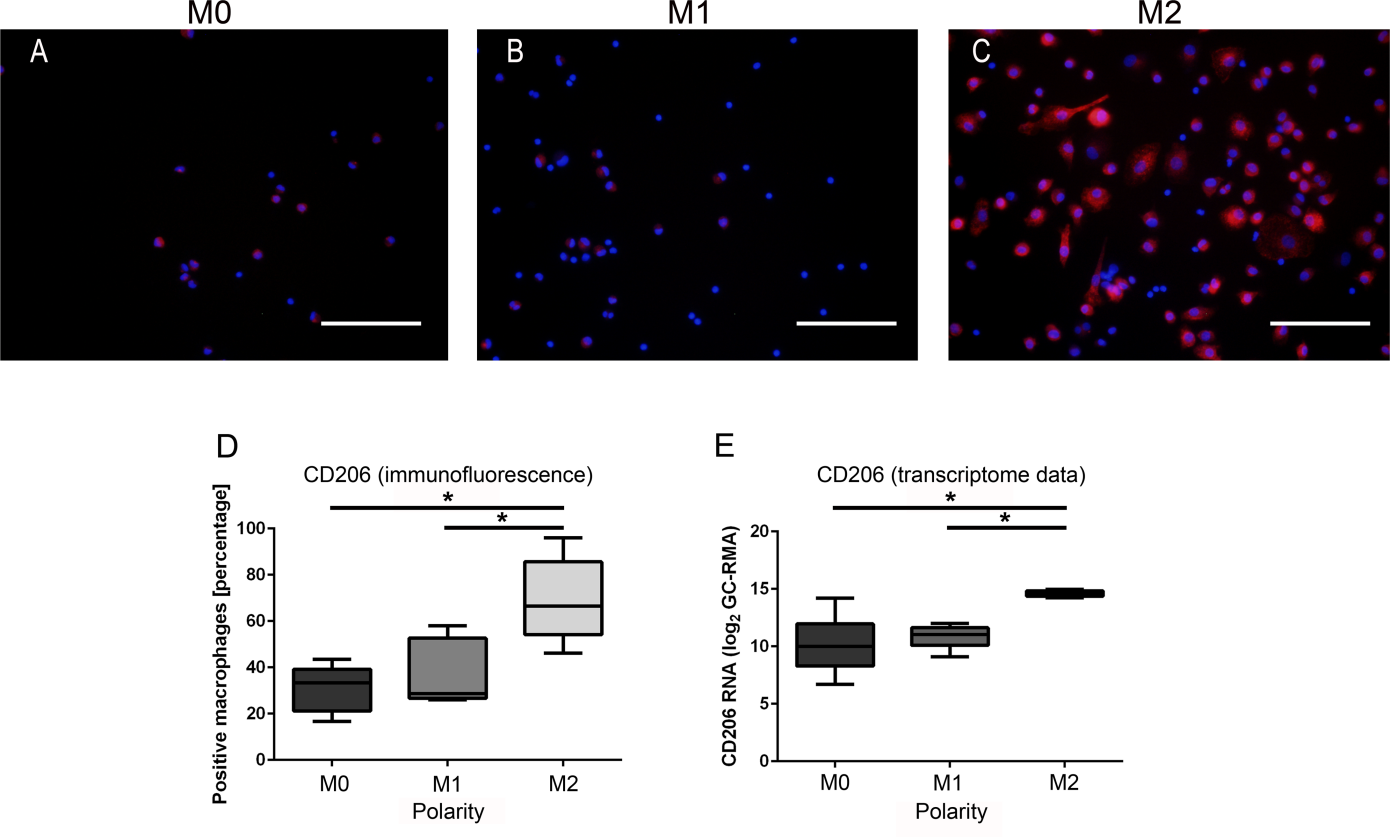
Phenotypical characterization of canine M0-, M1-, and M2-macrophages. **A)** Low membranous expression of CD206 antigen by small/roundish M0-macrophages. **B)** Moderate membranous staining of amoeboid M1-macrophages for CD206. **C)** Intense membranous expression of CD206 antigen by M2-macrophages. Scale bars = 100 μm. Nuclear counterstaining with bisbenzimide. **D)** The mean percentage of CD206-immunopositive cells is significantly higher in M2-macrophages as compared to both M1- and M0-macrophages (* = p≤0.05; Kruskal-Wallis-Test with pair-wise Mann-Whitney-U-Tests). **E)** The log_2_-transformed expression values of the probe set encoding for the gene CD206 is similarly significantly higher in M2-macrophages compared to both M1- and M0-macrophages (* = p≤0.05; Kruskal-Wallis-Test with pair-wise Mann-Whitney-U-Tests), thus confirming the results of the immunofluorescence investigation.

For CD206, the mean percentage of immunopositive cells was highest in M2-macrophages (mean: 66.54%) as compared to both M0-macrophages (mean: 33.33%; p = 0.008) and M1-macrophages (mean: 28.67%; p = 0.032, Fig 3). No statistical difference was observed between M1- and M0-macrophages (Fig 3). The results were validated in the microarray data set, which similarly revealed significantly higher expression levels of CD206 in canine M2-macrophages as compared to both M1- (p = 0.002) and M0-macrophages (p = 0.004; Fig 3).

### Differentially expressed genes between M0-, M1-, and M2-polarized macrophage cultures

One-factorial multigroup analysis of microarray data and fold change criteria identified 6358 probe sets that were differentially expressed in at least one of the three post-hoc pairwise comparisons. The total number of DEPs was 3555 in M1 *vs*. M0, 4831 in M2 *vs*. M0, and 3141 in M2 *vs*. M1, respectively (Table 2). In all pairwise comparisons, the number of up- and down-regulated probe sets was nearly equally distributed (M1 *vs*. M0: 1699 up, 1856 down; M2 *vs*. M0: 2467 up, 2364 down; M2 *vs*. M1: 1572 up, 1569 down). Functional annotation of enriched biological processes in DEPs, up–regulated in the comparison M1 *vs*. M0, revealed terms for “organonitrogen compound metabolic process”, “carbohydrate metabolic process”, “carboxylic acid metabolic process”, and “tricarboxylic acid cycle” (Table 2). In contrast, biological terms in down-regulated DEPs in this comparison reflected terms for “positive regulation of immune response”, “immune response-regulating signaling pathway”, and “regulation of innate immune response”. In both comparisons M2 *vs*. M0 and M2 *vs*. M1, up-regulated DEPs displayed significantly enriched gene ontology terms for “M phase of mitotic cell cycle” and “mitotic spindle organization”. Additionally, in M2 *vs*. M0 comparison, up-regulated DEPs were associated with “oxidation-reduction process”, “carboxylic acid metabolic process”, and “organonitrogen compound metabolic process”, whereas down-regulated DEPs were related to the biological term “immune response-activating signal transduction”. In the comparisons M2 *vs*. M1, down-regulated DEPs were functionally associated to biological terms such as “response to other organism”, “defense response”, “regulation of lymphocyte activation”, and “regulation of immune response” (Table 2).

**Table 2:** Summarized results of the functional annotation of the pairwise comparisons of differentially expressed probe sets (DEPs) in canine M0-, M1-, and M2-macrophages.

Pairwise comparison	Differentially expressed probe sets	Up-/down-regulated probe sets	Enriched biological process categories*	Enriched KEGG pathways*
M1 *vs*. M0	3555	Up: 1699	• Organonitrogen compound metabolic process • Carbohydrate metabolic process • Tricarboxylic acid cycle	• Metabolic pathways • Glutathione metabolism • Steroid biosynthesis • Glycolysis/Gluconeogenesis
		Down: 1856	• Positive regulation of immune response • Immune response-regulating signaling pathway • Regulation of innate immune response	• Hematopoietic cell lineage • RIG-I-like receptor signaling pathway • T cell receptor signaling pathway • Cell adhesion molecules
M2 *vs*. M0	4831	Up: 2467	• Oxidation-reduction process • Carboxylic acid metabolic process • Organonitrogen compound metabolic process • M-phase of mitotic cell cycle	• Metabolic pathways • Glycolysis/Gluconeogenesis • Propanoate and pyruvate metabolism • Steroid biosynthesis • Cell cycle
		Down: 2364	• Immune response-activating signal transduction	• T cell receptor signaling pathway • Natural killer cell mediated cytotoxicity • Hematopoietic cell lineage
M2 *vs*. M1	3141	Up: 1572	• Mitotic spindle organization	• Cell cycle • Metabolic pathways • Ribosome biogenesis in eukaryotes • PPAR signaling pathway
		Down: 1569	• Response to other organism • Defense response • Regulation of lymphocyte activation • Regulation of immune response	• NOD-like receptor signaling pathway • Osteoclast differentiation • Toll-like receptor signaling pathway • B and T cell receptor signaling pathway

* employing Web-based Gene Set Analysis Toolkit (WebGestalt; http://bioinfo.vanderbilt.edu/webgestalt/) with default settings

adjusted p-value ≤0.05

Enriched KEGG-pathways, significantly associated with up-regulated canine DEPs in M1 *vs*. M0 (1699 DEPs), were functionally related to “metabolic pathways”, “glutathione metabolism”, “steroid biosynthesis”, and “glycolysis/gluconeogenesis”. The comparison M2 *vs*. M0 (2467 up-regulated DEPs) contained enriched KEGG-pathways for “metabolic pathways”, “glycolysis/gluconeogenesis”, “propanoate and pyruvate metabolism”, “steroid biosynthesis”, and “cell cycle”. In the comparison M2 *vs*. M1 (1572 up-regulated DEPs), enriched KEGG-pathways included terms for “cell cycle”, “metabolic pathways”, “ribosome biogenesis in eukaryotes”, and “PPAR signaling pathway”. In contrast, down-regulated DEPs in the three comparisons were associated with “hematopoietic cell lineage”, “RIG-I-like receptor signaling pathway”, “T cell receptor signaling pathway”, and “cell adhesion molecules” (M1 *vs*. M0), “T cell receptor signaling pathway”, “natural killer cell mediated cytotoxicity”, and “hematopoietic cell lineage” (M2 *vs*. M0), and “NOD-like receptor signaling pathway”, “osteoclast differentiation”, “Toll-like receptor signaling pathway”, and “B and T cell receptor signaling pathway” (M2 *vs*. M1; Table 2).

Genes whose expression was most severely affected (FC ≥50.0 or ≤-50.0) in the pairwise comparison are depicted in Tables 3, 4 and 5. Fortynine genes fulfilled these filtering criteria in the M1 *vs*. M0 contrast (25 up, 24 down), whereas 99 genes were retrieved in the comparison of M2 *vs*. M0 (41 up; 58 down). Comparing M2 with M1, 66 genes fulfilled the criteria (32 up; 34 down). Focusing on potentially promising cell surface markers, the pairwise comparison of three genes encoding for such surface molecules were up-regulated in M1 *vs*. M0, namely *SUCNR1*, *SDC4*, and *CHRNA9*, whereas *CD209*, *CD180*, *KLRG1*, *COLEC12*, and *C3AR1* were down-regulated (Table 3). Comparing M2 *vs*. M0, nine cell surface makers were up-regulated, *i*.*e*. *LYVE1*, *SUCNR1*, *CD1e*, *MRC1*, *TSPAN7*, *JAM3*, *ANTXR1*, *MS4A2*, and *CLEC4G* (Table 4). In contrast, a total amount of 18 cell surface marker genes was down-regulated, namely *ITGB8*, *LY6E*, *TRDC*, *NCR3*, *SELL*, *SIGLEC1*, *KLRD1*, *KLRB1*, *MPP6*, *P2RY14*, *TRBC2*, *TARP*, *FCRLA*, *KCNK5*, *P2RX5*, *NKG7*, *CD69*, and *CD7* (Table 4). In the comparison M2 *vs*. M1, the up-regulated cell surface marker genes contained 10 terms for *FCER1A*, *CD209*, *LYVE1*, *CLEC4G*, *COLEC12*, *JAM3*, *MS4A2*, *STAB1*, *MRC1*, and *SLC15A1* (Table 5). Down-regulated cell surface marker genes were *P2RY14*, *SLC39A14*, *ITGB8*, *TMEM150C*, *TMEM176A*, and *SLC22A15* (Table 5).

**Table 3:** List and subgrouping of the top hits of highly differentially expressed genes (fold change ≥ 50 or ≤ -50) in canine M1- *vs*. M0-macrophages.

Gene name	Gene symbol	Fold change
***Up-regulated genes***		
***Cell surface markers***		
Succinate receptor 1	SUCNR1	212.94
Syndecan 4	SDC4	58.61
Cholinergic receptor, nicotinic, alpha 9 (neuronal)	CHRNA9	53.74
***Enzymes***		
ADP-ribosylhydrolase like 2	ADPRHL2	535.59
Ceruloplasmin (ferroxidase)	CP	161.88
Epoxide hydrolase 2, cytoplasmic	EPHX2	119.72
Ectonucleotide pyrophosphatase/phosphodiesterase 2	ENPP2	117.43
Interstitial collagenase-like	LOC489428	87.83
E3 ubiquitin-protein ligase NEURL3-like	LOC102152163	86.20
NOP2/Sun domain family, member 7	NSUN7	66.00
Nucleoredoxin	NXN	64.78
WNK lysine deficient protein kinase 2	WNK2	52.35
***Cytokines*, *chemokines*, *and their Receptors***		
Interleukin 6 (interferon, beta 2)	IL6	252.32
Chemokine (C-C motif) ligand 22	CCL22	187.96
Chemokine (C-X-C motif) receptor 7	ACKR3	178.18
Interleukin 22 receptor, alpha 2	IL22RA2	116.00
Chemokine (C-X-C motif) ligand 14	CXCL14	108.55
Chemokine (C-C motif) ligand 17	CCL17	95.26
Chemokine (C-C motif) ligand 20	CCL20	67.90
***Soluble factors***		
Chitinase 3-like 1 (cartilage glycoprotein-39)	CHI3L1	83.25
Clusterin	CLU	66.23
***Miscellaneous***		
Ras homolog family member U	RHOU	446.23
Interferon, alpha-inducible protein 6	IFI6	269.13
CXADR-like membrane protein	CLMP	213.81
Retinoic acid induced 14	RAI14	118.86
***Down-regulated genes***		
***Cell surface markers***		
CD209 molecule	CD209	-345.12
CD180 molecule	CD180	-75.00
Killer cell lectin-like receptor subfamily G, member 1	KLRG1	-66.25
Collectin sub-family member 12	COLEC12	-61.81
Complement component 3a receptor 1	C3AR1	-55.16
***Enzymes***		
Carboxypeptidase M	CPM	-134.50
N-acetylneuraminate pyruvate lyase (dihydrodipicolinate synthase)	NPL	-78.98
Cathepsin E	CTSE	-76.30
***Cytokines*, *chemokines*, *and their receptors***		
Chemokine (C-X-C motif) ligand 12	CXCL12	-401.92
Pro-platelet basic protein (chemokine (C-X-C motif) ligand 7)	PPBP	-100.42
Interleukin 2	IL2	-95.84
Interleukin 1 receptor, type II	IL1R2	-79.71
Chemokine (C-C motif) receptor 3	CCR3	-51.05
***Soluble factors***		
Lipocalin 2	LCN2	-180.73
CD5 molecule-like	CD5L	-153.48
Secreted phosphoprotein 2, 24kDa	SPP2	-51.92
***Miscellaneous***		
Coagulation factor XIII, A1 polypeptide	F13A1	-3305.03
Fatty acid binding protein 4, adipocyte	FABP4	-370.34
G protein-coupled receptor 116	GPR116	-130.89
Interferon-induced transmembrane protein 3-like	LOC606890	-114.16
Plexin domain containing 2	PLXDC2	-67.07
ADP-ribosylation factor-like 4C	ARL4C	-56.52
Cyclin J-like	CCNJL	-54.76
Thrombospondin 1	THBS1	-53.38

**Table 4:** List and subgrouping of the top hits of highly differentially expressed genes (fold change ≥ 50 or ≤ -50) in canine M2- *vs*. M0-macrophages.

Gene name	Gene symbol	Fold change
***Up-regulated genes***		
***Cell surface markers***		
Lymphatic vessel endothelial hyaluronan receptor 1	LYVE1	322.32
Succinate receptor 1	SUCNR1	151.91
CD1e molecule	CD1E	147.40
Mannose receptor, C type 1	MRC1	104.41
Tetraspanin 7	TSPAN7	83.25
Junctional adhesion molecule 3	JAM3	78.46
Anthrax toxin receptor 1	ANTXR1	72.95
Membrane-spanning 4-domains, subfamily A, member 2	MS4A2	54.98
C-type lectin domain family 4, member G	CLEC4G	54.14
***Enzymes***		
ADP-ribosylhydrolase like 2	ADPRHL2	614.52
Guanine deaminase	GDA	174.44
Ubiquitin-conjugating enzyme E2 C-like	LOC481325	151.23
Ribonucleotide reductase M2	RRM2	131.03
Fructose-1,6-bisphosphatase 1	FBP1	57.95
Matrix metallopeptidase 9 (gelatinase B, 92kDa gelatinase, 92kDa type IV collagenase)	MMP9	56.03
Lipoprotein lipase	LPL	52.03
Trimethyllysine hydroxylase, epsilon	TMLHE	51.05
3-hydroxybutyrate dehydrogenase, type 2	BDH2	50.68
***Cytokines*, *chemokines*, *and their receptors***		
Chemokine (C-C motif) ligand 24	CCL24	1060.82
Chemokine (C-X-C motif) receptor 7	ACKR3	390.67
Chemokine (C-C motif) ligand 17	CCL17	209.36
Chemokine (C-C motif) ligand 13	CCL13	172.58
Interleukin 13 receptor, alpha 2	IL13RA2	170.59
Transforming growth factor, beta 2	TGFB2	61.84
***Soluble factors***		
Nephronectin	NPNT	444.07
Norrie disease (pseudoglioma)	NDP	213.72
Endothelin 1	EDN1	115.81
Cystatin 9 (testatin)	CST9	58.21
***Miscellaneous***		
Sodium channel, voltage-gated, type II, beta subunit	SCN2B	1061.28
Caldesmon 1	CALD1	224.66
CXADR-like membrane protein	CLMP	169.27
SHC SH2-domain binding protein 1	SHCBP1	117.77
Dynamin 1	DNM1	112.78
NACC family member 2, BEN and BTB (POZ) domain containing	NACC2	110.49
Retinoic acid induced 14	RAI14	84.35
Scinderin	SCIN	71.66
Kinesin family member 23	KIF23	66.78
Kinesin family member 11	KIF11	64.20
Cfa-mir-125b-2	cfa-mir-125b-2	58.78
AHNAK nucleoprotein	AHNAK	57.19
NUF2, NDC80 kinetochore complex component	NUF2	54.62
***Down-regulated genes***		
***Cell surface markers***		
Integrin, beta 8	ITGB8	-199.37
Lymphocyte antigen 6 complex, locus E	LY6E	-116.70
T cell receptor delta constant	TRDC	-112.73
Natural cytotoxicity triggering receptor 3	NCR3	-105.99
Selectin L	SELL	-95.45
Sialic acid binding Ig-like lectin 1, sialoadhesin	SIGLEC1	-91.39
Killer cell lectin-like receptor subfamily D, member 1	KLRD1	-87.12
Killer cell lectin-like receptor subfamily B, member 1	KLRB1	-74.56
Membrane protein, palmitoylated 6 (MAGUK p55 subfamily member 6)	MPP6	-72.60
Purinergic receptor P2Y, G-protein coupled, 14	P2RY14	-71.69
T cell receptor beta constant 2	TRBC2	-64.26
TCR gamma alternate reading frame protein	TARP	-62.39
Fc receptor-like A	FCRLA	-60.06
Potassium channel, subfamily K, member 5	KCNK5	-58.16
Purinergic receptor P2X, ligand-gated ion channel, 5	P2RX5	-58.16
Natural killer cell group 7 sequence	NKG7	-57.26
CD69 molecule	CD69	-54.53
CD7 molecule	CD7	-52.57
***Enzymes***		
Interferon stimulated exonuclease gene 20kDa	ISG20	-514.04
Prostaglandin E synthase	PTGES	-382.20
Cytidine monophosphate (UMP-CMP) kinase 2, mitochondrial	CMPK2	-266.50
Granzyme A (granzyme 1, cytotoxic T-lymphocyte-associated serine esterase 3)	GZMA	-131.27
Granzyme B (granzyme 2, cytotoxic T-lymphocyte-associated serine esterase 1)	GZMB	-130.63
Hexokinase 3 (white cell)	HK3	-127.10
Ubiquitin specific peptidase 18	USP18	-81.72
Phospholipase A1 member A	PLA1A	-72.65
Cathepsin E	CTSE	-72.13
GTP cyclohydrolase 1	GCH1	-66.29
Chymase 1, mast cell	CMA1	-61.45
Phospholipid scramblase 1-like	LOC611500	-61.09
***Cytokines*, *chemokines*, *and their receptors***		
Chemokine (C-X-C motif) ligand 12	CXCL12	-417.05
Interleukin 2	IL2	-195.40
Interleukin 7 receptor	IL7R	-66.71
Transforming growth factor, beta receptor III	TGFBR3	-50.94
***Soluble factors***		
Lipocalin 2	LCN2	-217.74
Adrenomedullin	ADM	-100.37
***Miscellaneous***		
Radical S-adenosyl methionine domain containing 2	RSAD2	-559.34
Interferon-induced transmembrane protein 3-like	LOC606890	-435.57
ISG15 ubiquitin-like modifier	ISG15	-254.32
Apolipoprotein L, 5	APOL5	-200.23
Fatty acid binding protein 4, adipocyte	FABP4	-182.17
Interferon-induced protein with tetratricopeptide repeats 1	IFIT1	-142.72
Interferon regulatory factor 4-like	LOC609817	-118.68
Carcinoembryonic antigen-related cell adhesion molecule 25	CAECAM1	-113.27
Interferon regulatory factor 7	IRF7	-102.93
OCIA domain containing 2	OCIAD2	-100.84
Piwi-like RNA-mediated gene silencing 4	PIWIL4	-97.75
Synaptotagmin-like 3	SYTL3	-94.94
Testis expressed 14	TEX14	-92.58
Myxovirus (influenza virus) resistance 1, interferon-inducible protein p78 (mouse)	MX1	-91.32
Structural maintenance of chromosomes flexible hinge domain containing 1	SMCHD1	-84.97
Interferon-induced transmembrane protein 1-like	LOC475935	-83.91
eukaryotic peptide chain release factor GTP-binding subunit ERF3B-like	LOC480921	-74.06
Lactotransferrin	LTF	-72.08
DEAD (Asp-Glu-Ala-Asp) box polypeptide 58	DDX58	-69.54
TNFAIP3 interacting protein 3	TNIP3	-58.03
Syntrophin, beta 1 (dystrophin-associated protein A1, 59kDa, basic component 1)	SNTB1	-55.46
Src kinase associated phosphoprotein 1	SKAP1	-50.58

**Table 5:** List and subgrouping of the top hits of highly differentially expressed genes (fold change ≥ 50 or ≤ -50) in M2- *vs*. M1-macrophages.

Gene name	Gene symbol	Fold change
***Up-regulated genes***		
***Cell surface markers***		
Fc fragment of IgE, high affinity I, receptor for; alpha polypeptide	FCER1A	376.89
CD209 molecule	CD209	335.12
Lymphatic vessel endothelial hyaluronan receptor 1	LYVE1	321.11
C-type lectin domain family 4, member G	CLEC4G	183.13
Collectin sub-family member 12	COLEC12	181.04
Junctional adhesion molecule 3	JAM3	145.24
Membrane-spanning 4-domains, subfamily A, member 2	MS4A2	137.95
Stabilin 1	STAB1	127.60
Mannose receptor, C type 1	MRC1	52.68
Solute carrier family 15 (oligopeptide transporter), member 1	SLC15A1	52.03
***Enzymes***		
Fructose-1,6-bisphosphatase 1	FBP1	149.73
Uronyl-2-sulfotransferase	UST	85.20
Alanyl (membrane) aminopeptidase	ANPEP	82.13
Lipoprotein lipase	LPL	57.39
***Cytokines*, *chemokines*, *and their receptors***		
Chemokine (C-C motif) ligand 24	CCL24	1050.11
Transforming growth factor, beta 2	TGFB2	66.97
***Soluble factors***		
CD5 molecule-like	CD5L	209.01
Secreted phosphoprotein 2, 24kDa	SPP2	121.14
Nephronectin	NPNT	105.07
Secretogranin V (7B2 protein)	SCG5	82.69
Complement component 3	C3	67.78
Cystatin 9 (testatin)	CST9	58.53
***Miscellaneous***		
Coagulation factor XIII, A1 polypeptide	F13A1	1309.86
Sodium channel, voltage-gated, type II, beta subunit	SCN2B	1120.45
Dynamin 1	DNM1	247.10
Caldesmon 1	CALD1	239.74
Rho GTPase activating protein 6	ARHGAP6	147.58
Fibronectin 1	FN1	141.81
Cfa-mir-125b-2	cfa-mir-125b-2	103.61
Plexin domain containing 2	PLXDC2	71.70
Transforming growth factor, beta-induced, 68kDa	TGFBI	59.20
G protein-coupled receptor 116	GPR116	51.83
***Down-regulated genes***		
***Cell surface markers***		
Purinergic receptor P2Y, G-protein coupled, 14	P2RY14	-344.22
Solute carrier family 39 (zinc transporter), member 14	SLC39A14	-200.01
Integrin, beta 8	ITGB8	-134.58
Transmembrane protein 150C	TMEM150C	-103.02
Transmembrane protein 176A	TMEM176A	-78.17
Solute carrier family 22, member 15	SLC22A15	-58.69
***Enzymes***		
Prostaglandin E synthase	PTGES	-1274.66
Epoxide hydrolase 2, cytoplasmic	EPHX2	-222.01
Interferon stimulated exonuclease gene 20kDa	ISG20	-187.95
Prostaglandin-endoperoxide synthase 2 (prostaglandin G/H synthase and cyclooxygenase)	PTGS2	-183.48
E3 ubiquitin-protein ligase NEURL3-like	LOC102152163	-156.11
Indoleamine 2,3-dioxygenase 2	IDO2	-127.88
STEAP family member 4	STEAP4	-92.42
Ceruloplasmin (ferroxidase)	CP	-90.30
Granzyme B (granzyme 2, cytotoxic T-lymphocyte-associated serine esterase 1)	GZMB	-69.42
Phospholipase A2, group XVI-like	LOC476045	-57.39
cytidine monophosphate (UMP-CMP) kinase 2, mitochondrial	CMPK2	-57.00
WNK lysine deficient protein kinase 2	WNK2	-52.30
Interleukin-1 receptor-associated kinase 3	IRAK3	-50.40
***Cytokines*, *chemokines*, *and their receptors***		
Chemokine (C-X-C motif) receptor 3	CXCR3	-140.97
Chemokine (C-C motif) ligand 20	CCL20	-119.64
Interleukin 6 (interferon, beta 2)	IL6	-69.50
Tumor necrosis factor	TNF	-55.63
***Soluble factors***		
Complement component 2	C2	-78.67
***Miscellaneous***		
Peptidase inhibitor 3, skin-derived	PI3	-235.52
TNFAIP3 interacting protein 3	TNIP3	-178.37
Ras homolog family member U	RHOU	-168.17
Radical S-adenosyl methionine domain containing 2	RSAD2	-82.43
Cochlin	COCH	-70.23
Interferon, alpha-inducible protein 6	IFI6	-68.46
multiple C2 domains, transmembrane 2	MCTP2	-66.09
Fascin homolog 1, actin-bundling protein (Strongylocentrotus purpuratus)	FSCN1	-58.64
Interferon regulatory factor 4-like	LOC609817	-50.92
ISG15 ubiquitin-like modifier	ISG15	-50.28

Interestingly, gene expression of *CD209* markedly decreased in the pairwise comparison of M1 *vs*. M0 (FC = -345.12) whereas it was highly up-regulated in the comparison M2 *vs*. M1 (FC = 335.12). In both comparisons, M2 *vs*. M0 and M2 *vs*. M1, genes for *LYVE1*, *MRC1*, *MS4A2*, *JAM3*, and *CLEC4G* were up-regulated, whereas genes for *ITGB8* and *P2RY14* were down-regulated. *SUCNR1* was up-regulated in both M1 *vs*. M0 and M2 *vs*. M0.

### Hierarchical cluster analysis

**Fig 4:**
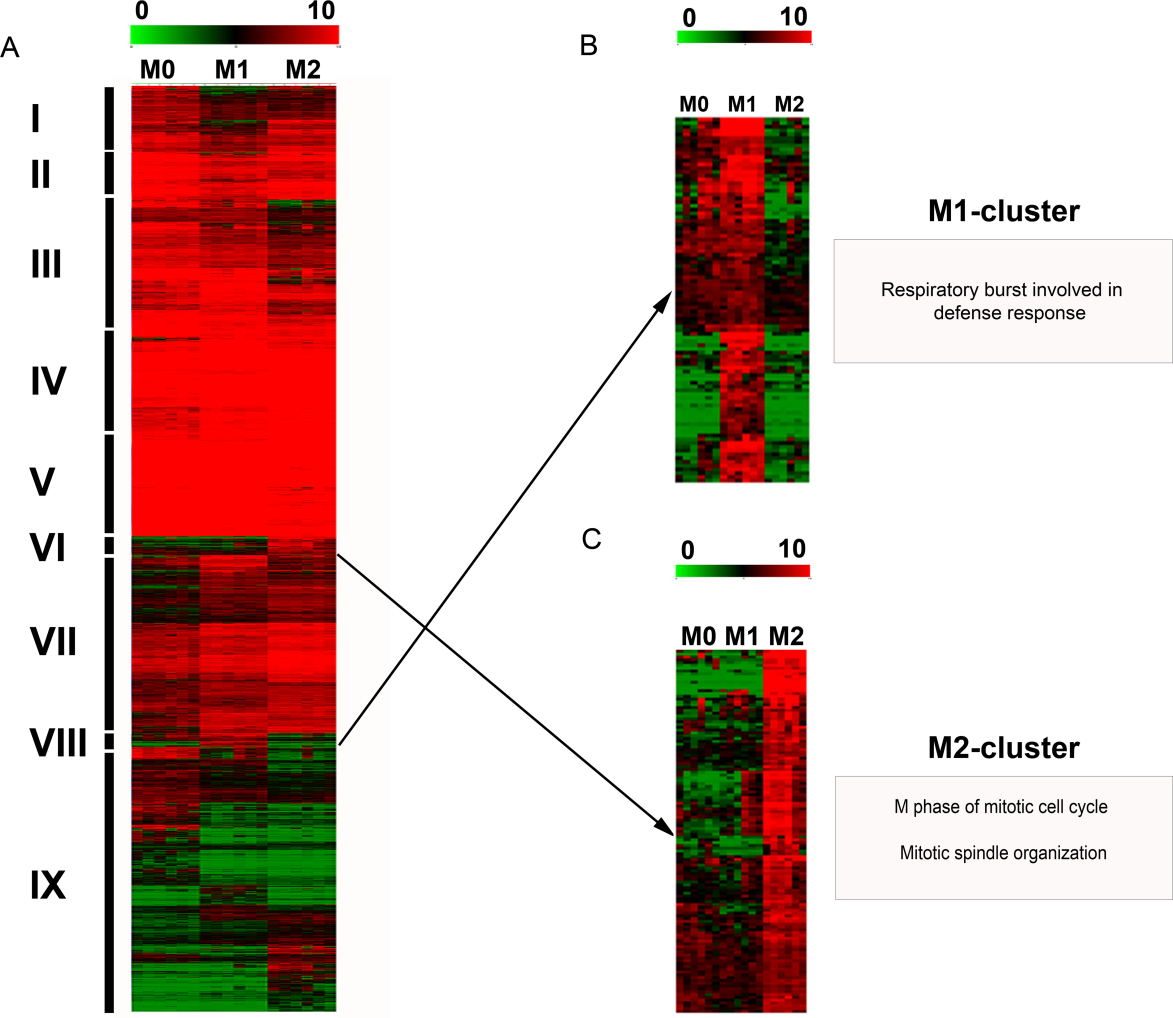
Hierarchical clustering analysis. Unsupervised hierarchical clustering analysis of the median centered, log_2_-transformed expression values of 6358 differentially expressed probe sets in canine polarized macrophages as obtained by TM4 MultiExperimentViewer with default settings (Euclidean distance; complete linkage), depicted on a color scale from red (relatively high expression) to green (relatively low expression). **A)** The analysis identified 9 distinct clusters (I-IX) based on similarities as well as differences in the expression intensity of canine polarized macrophages. Two out of these 9 clusters (VIII and VI) visually displayed an expression profile that was clearly associated with either the M1- or M2-phenotype. **B)** Functional annotation of the M1-cluster (magnified from A) using Web-based Gene Set Analysis Toolkit (WebGestalt) identified the enriched biological process category “respiratory burst” (adjusted p-value≤0.05). **C)** The M2-cluster (magnified from A) is associated with enriched biological GO terms such as “M phase of mitotic cell cycle” and “mitotic spindle organization” (adjusted p-value≤0.05).

Unsupervised hierarchical clustering analysis formed 9 different clusters based on similarities and differences in the expression profile of DEPs (Fig 4). Two out of these 9 clusters had an expression profile that was visually clearly associated with either the M1- or M2-phenotype (Fig 4). Functional annotation of these clusters identified that the M1-polarization cluster (cluster 8) was significantly associated with the biological process “respiratory burst involved in defense response” (Fig 4). The M2-cluster (cluster 6) was significantly associated with multiple biological processes of mitosis such as “M phase of mitotic cell cycle” and “mitotic spindle organization” (Fig 4). The genes associated with these M1- and M2-specific clusters are listed with their particular fold changes in S1 and S2 Tables. The remaining 7 clusters were neither specific for M1- nor for M2-macrophages and were associated with biological terms like “peptidyl-lysine mono- and dimethylation” (cluster 1), “immune response-activating signal transduction” (cluster 3), “monosaccharide metabolic process”, “organic substance catabolic process”, “cellular catabolic process” (cluster 4), “response to other organism”, “regulation of lymphocyte proliferation” (cluster 5), “tRNA aminoacylation for protein translation” (cluster 7), “cytokinesis” and “antigen receptor-mediated signaling pathway” (cluster 9, S3 Table). No significantly enriched biological terms were identified for cluster 2.

### Literature-based gene signatures and marker genes

**Fig 5:**
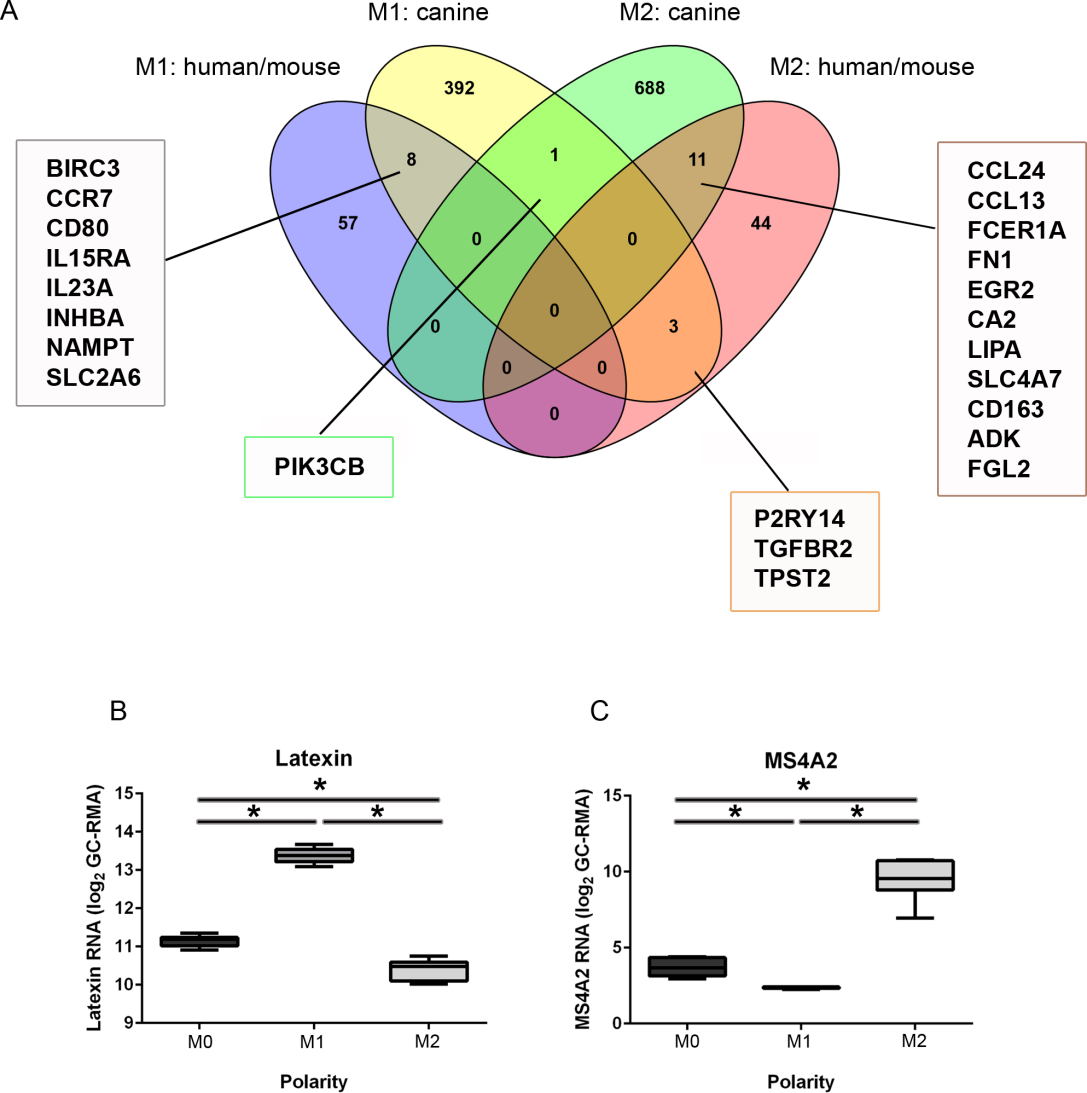
**Comparative evaluation of canine M1- and M2-associated differentially expressed genes (M1 = 404; M2 = 700) with established literature-based human and murine orthologous genes (A) and detection of polarization-specific biomarkers employing supervised clustering with a K-nearest-neighbors algorithm and correlation-based feature selection using Prophet (B, C). A)** The Venn diagram depicts the numbers and intersections of differential and common canine M1- and M2-genes with literature-based human and murine genes. The majority of literature-based M1- (65 genes = 57+8) and M2-markers (58 genes = 44+11+3) did not comply with the present microarray data upon canine macrophages. However, overlapping expression of 8 genes for the M1-phenotype and 11 genes for the M2-phenotype was identified. The genes of the intersections, not specifically mentioned in the figure, are listed in S4 Table **B)** Biomarker selection detected 4 highly specific probe sets for annotated by the mammalian carboxypeptidase inhibitor *latexin* (*LXN*) to be highly correlated to the M1-phenotype. The boxplots depict the significantly enhanced log_2_-transformed expression values for *LXN* in M1-macrophages as compared to both M0- and M2-macrophages (p = 0.002), as well as between M0- and M2-macrophages (p = 0.002). **C)** For the M2-phenotype, the most significant predicted biomarker was high affinity receptor for IgE, *i*.*e*. membrane-spanning 4-domains, subfamily A, member 2 (*MS4A2*). The expression data of M2-macrophages show significantly higher log_2_-transformed expression values as compared to both M0- and M1-macrophages (p = 0.002) as well as between M0- and M1-macrophages (p = 0.002). Asterisks indicate significance (Kruskal-Wallis-Test with subsequent pairwise Mann-Whitney-U-Tests).

Intersections of genes exclusively up-regulated in M1 *vs*. M0 and down regulated in M2 *vs*. M1 (M1-macrophage genes), as well as genes up-regulated in the comparisons M2 *vs*. M0 and M2 *vs*. M1 (M2-macrophage genes) were selected and compared to literature-based markers that are known to distinguish between human and murine M1- and M2-macrophages (Fig 5A). Notably, many markers identified in the recent study to be specific for canine M1-polarization (404 unique genes in total corresponding to 565 probesets, S5 Table) did not match with the literature-based M1-markers (65 genes). Similarly, predominating numbers of canine M2-markers (700 unique genes in total corresponding to 1029 probesets, S5 Table) did not match with the literature-based M2-markers (58 genes; Fig 5A). However, overlapping expression of genes reflecting M1-polarization state was present for 8 genes, *i*.*e*. *BIRC3*, *CCR7*, *CD80*, *IL15RA*, *IL23A*, *INHBA*, *NAMPT*, and *SLC2A6*. For canine M2-polarization, 11 genes matched reported expression in human and murine M2-macrophages, *i*.*e*. *CCL24*, *CCL13*, *FCER1A*, *FN1*, *EGR2*, *CA2*, *LIPA*, *SLC4A7*, *CD163*, *ADK*, and *FGL2* (Fig 5A). Conflicting results were present for the genes *P2RY14*, *TGFBR2*, and *TPST2*, which were expected as M2-markers based on the literature but were differentially up-regulated in canine M1-macrophages. Furthermore, *PIK3CB* was up-regulated by both canine M1- and M2-macrophages in the present study. The differentially expressed genes of the intersections, which are not namely mentioned in Fig 5A are listed in S4 Table. The top 50 candidate canine M1- and M2-macrophage genes, as defined above, are given in Tables 6 and 7. The full lists of canine probesets, corresponding to the 404 unique M1 genes and 700 unique M2 genes are shown in S5 Table.

**Table 6:** Top 50 candidate M1-macrophage associated probesets, which were upregulated in M1- vs. M0-macrophages and simultaneously downregulated in M2- vs. M1-macrophages.

Probe Set ID	Gene Symbol	Fold change M1 vs. M0	Fold change M2 vs. M1
Cfa.12190.1.A1_at	PTGES	3.34	-1274.66
CfaAffx.30585.1.S1_s_at	PTGES	4.99	-1136.51
CfaAffx.30585.1.S1_at	PTGES	4.94	-517.22
CfaAffx.927.1.S1_at	P2RY14	4.80	-344.22
Cfa.20456.1.S1_at	IFI6	269.13	-284.48
CfaAffx.15155.1.S1_s_at	PI3	27.22	-235.52
Cfa.574.1.A1_at	EPHX2	119.72	-222.01
Cfa.18083.1.S1_s_at	SLC39A14	15.89	-212.19
CfaAffx.14855.1.S1_s_at	SLC39A14	13.48	-200.01
Cfa.3449.1.S1_s_at	PTGS2	27.78	-183.48
Cfa.15554.1.A1_at	TNIP3	3.07	-178.37
Cfa.9253.1.A1_at	RHOU	446.23	-168.17
Cfa.12477.1.A1_at	LOC102152163	86.20	-156.11
Cfa.16339.1.S1_at	CXCR3	5.70	-140.97
Cfa.10779.1.A1_at	IDO2	28.65	-127.88
CfaAffx.13394.1.S1_s_at	EPHX2	110.24	-122.12
Cfa.15812.1.S1_at	CCL20	67.90	-119.64
Cfa.1856.1.S1_at	TMEM150C	40.36	-103.02
Cfa.2878.1.A1_s_at	CP	425.52	-96.02
CfaAffx.3697.1.S1_at	STEAP4	11.45	-92.42
CfaAffx.13209.1.S1_s_at	CP	161.88	-90.30
Cfa.8846.1.A1_s_at	C2 /// CFB	4.39	-78.67
CfaAffx.7919.1.S1_at	TMEM176A	19.58	-78.17
CfaAffx.1718.1.S1_at	TNF	47.17	-71.34
Cfa.3528.1.S1_s_at	IL6	252.32	-69.50
Cfa.20456.1.S1_s_at	IFI6	68.64	-68.46
CfaAffx.17110.1.S1_s_at	MCTP2	2.69	-66.09
CfaAffx.15348.1.S1_at	SLC22A15	15.67	-58.69
CfaAffx.24565.1.S1_at	FSCN1	35.83	-58.64
Cfa.12164.1.A1_at	LOC476045	5.65	-57.39
Cfa.54.1.S1_s_at	TNF	47.20	-55.63
Cfa.11870.1.A1_at	PI3	17.25	-52.33
Cfa.4359.1.S1_at	WNK2	52.35	-52.30
CfaAffx.1510.1.S1_s_at	IRAK3	10.70	-50.40
CfaAffx.15202.1.S1_s_at	SDC4	58.61	-49.56
CfaAffx.1352.1.S1_s_at	IL22RA2	116.00	-48.48
CfaAffx.26233.1.S1_s_at	CXCR3	32.55	-47.23
CfaAffx.17136.1.S1_s_at	MCTP2	6.25	-46.94
CfaAffx.24086.1.S1_at	KMO	23.66	-46.48
CfaAffx.15086.1.S1_s_at	BMP1	43.14	-43.31
CfaAffx.261.1.S1_at	HCAR3	13.88	-42.20
Cfa.18962.1.S1_s_at	SGK1	20.11	-39.12
Cfa.3719.1.S1_s_at	QPCT	22.78	-38.99
Cfa.4926.1.A1_s_at	LOC476045	5.41	-37.14
CfaAffx.6260.1.S1_at	C18H7orf10	43.19	-36.13
Cfa.6458.1.A1_s_at	C2 /// CFB	5.05	-36.01
Cfa.8282.1.A1_s_at	KBTBD7	3.64	-35.38
CfaAffx.8143.1.S1_at	KBTBD7	2.59	-35.14
Cfa.21252.1.S1_s_at	ENPP2	188.74	-34.85
CfaAffx.16422.1.S1_s_at	CCL20	33.43	-34.83

**Table 7:** Top 50 candidate M2-macrophage associated probesets, which were upregulated in M2- vs. M0-macrophages and simultaneously upregulated in M2- vs. M1-macrophages.

Probe Set ID	Gene Symbol	Fold change M2 vs. M0	Fold change M2 vs. M1
Cfa.11125.1.A1_at	SCN2B	1061.28	1120.45
Cfa.15823.1.S1_at	CCL24	1060.82	1050.11
CfaAffx.18273.1.S1_at	FCER1A	35.63	922.72
CfaAffx.20721.1.S1_s_at	CCL24	819.06	819.06
Cfa.17541.1.S1_s_at	FBP1	160.02	748.69
CfaAffx.2850.1.S1_s_at	FBP1	57.95	422.21
Cfa.3663.1.A1_s_at	FCER1A	21.07	376.89
Cfa.3707.1.A1_at	FN1	12.68	360.42
CfaAffx.12229.1.S1_at	LYVE1	322.32	321.11
Cfa.10966.1.A1_at	DNM1	112.78	247.10
Cfa.6272.1.S1_at	CALD1	224.66	239.74
CfaAffx.1504.1.S1_s_at	UST	7.71	226.47
Cfa.2693.1.A1_at	LOC102152647	194.28	194.28
Cfa.3707.1.A1_s_at	FN1	7.26	188.32
CfaAffx.28024.1.S1_at	CLEC4G	54.14	183.13
CfaAffx.16206.1.S1_at	MS4A2	110.05	167.77
Cfa.6369.1.A1_at	FBP1	26.98	149.73
Cfa.20346.1.S1_at	ARHGAP6	37.99	147.58
Cfa.1705.1.A1_at	JAM3	78.46	145.24
Cfa.3662.1.S1_at	MS4A2	54.98	137.95
Cfa.19567.1.S1_at	UST	8.05	120.81
CfaAffx.16206.1.S1_s_at	MS4A2	76.56	108.61
Cfa.3774.1.A1_s_at	ANPEP	25.55	105.73
Cfa.14465.1.S1_at	NPNT	444.07	105.07
Cfa.688.1.S1_at	cfa-mir-125b-2	58.78	103.61
CfaAffx.7700.1.S1_s_at	MRC1	136.99	89.14
Cfa.2468.1.A1_at	UST	6.54	85.20
Cfa.10794.1.A1_at	SCG5	6.75	82.69
Cfa.20798.1.S1_at	ANPEP	14.09	82.13
CfaAffx.14701.1.S1_s_at	STAB1	4.00	81.41
CfaAffx.28463.1.S1_at	C3	36.53	76.45
Cfa.12240.1.A1_at	C3	44.14	67.78
CfaAffx.16904.1.S1_s_at	TGFB2	61.84	66.97
CfaAffx.7365.1.S1_at	CST9	58.21	58.53
CfaAffx.15901.1.S1_s_at	LPL	52.03	57.39
CfaAffx.7698.1.S1_at	MRC1	104.41	52.68
Cfa.78.1.S1_s_at	SLC15A1	14.65	52.03
CfaAffx.27914.1.S1_s_at	IL13RA2	170.59	46.70
Cfa.19638.1.S1_s_at	TMLHE	51.05	45.55
CfaAffx.22229.1.S1_at	GPR34	6.30	42.39
CfaAffx.21392.1.S1_s_at	FKBP7	25.81	40.60
CfaAffx.19151.1.S1_s_at	ADCY4	14.07	39.02
Cfa.11222.1.A1_s_at	FAM185A	23.07	38.03
Cfa.10053.1.A1_at	RAB40B	48.86	37.88
CfaAffx.24797.1.S1_at	EPHX1	3.75	36.82
Cfa.1930.1.S1_at	MRC1	25.70	36.76
Cfa.3367.1.A1_at	TGFB2	36.49	36.58
Cfa.15824.1.S1_at	CCL13	172.58	34.71
Cfa.14297.1.A1_s_at	MAOB	33.75	33.75
Cfa.12621.1.A1_at	LRP4	7.01	33.01

### Biomarker selection

In a hypothesis-driven approach, polarization specific prediction markers were detected with Prophet using the microarray data of unstimulated macrophages (M0), M1-, and M2-macrophages [41, 42]. 369 probe sets were identified by the correlation-based feature selection algorithm of Prophet that discriminated between M0-, M1-, and M2-polarity, using the KNN algorithm with 100% correct predictions. Subsequent “one *versus* all” analyses using SET to create ranked lists of genes, based on their potential to serve as biomarker for M1- or M2-macrophages were performed (S6 Table; [43]). The highest scoring probe set differentiating M1- from M0- and M2-macrophages was CfaAffx.14358.1.S1_at, annotated as *latexin* (*LXN*), which was up-regulated in M1-macrophages, exhibiting a prediction-accuracy for the M1-phenotype of 100%. Additionally, the probe set ID’s occupying the ranks 2 to 4 (Cfa.5195.1.A1_s_at, Cfa.5195.1.A1_at, Cfa.14007.1.A1_x_at) were similarly annotated to *LXN* (S6 Table). Analysis of the expression data of CfaAffx.14358.1.S1_at revealed a relatively high expression in all three polarities with a significantly higher expression in M1-macrophages as compared to M0- and M2-macrophages (pairwise Mann-Whitney-U-Tests; p = 0.02, Fig 5B). For the comparison of M2 *versus* M0 and M1, the highest scoring probe set was Cfa.3662.1.S1_at, annotated as *membrane-spanning 4-domains*, *subfamily A*, *member 2* (*MS4A2*, S6 Table). This probe set was up-regulated in M2-macrophages and displayed a prediction accuracy of 100% for this phenotype. Statistical evaluation with pairwise Mann-Whitney-U-Tests underlined a low expression of *MS4A2* in M0- and M1-macrophages, but a significantly higher expression in M2-macrophages (p = 0.02, Fig 5C).

### Testing of antibodies for the detection of predicted biomarkers

**Fig 6:**
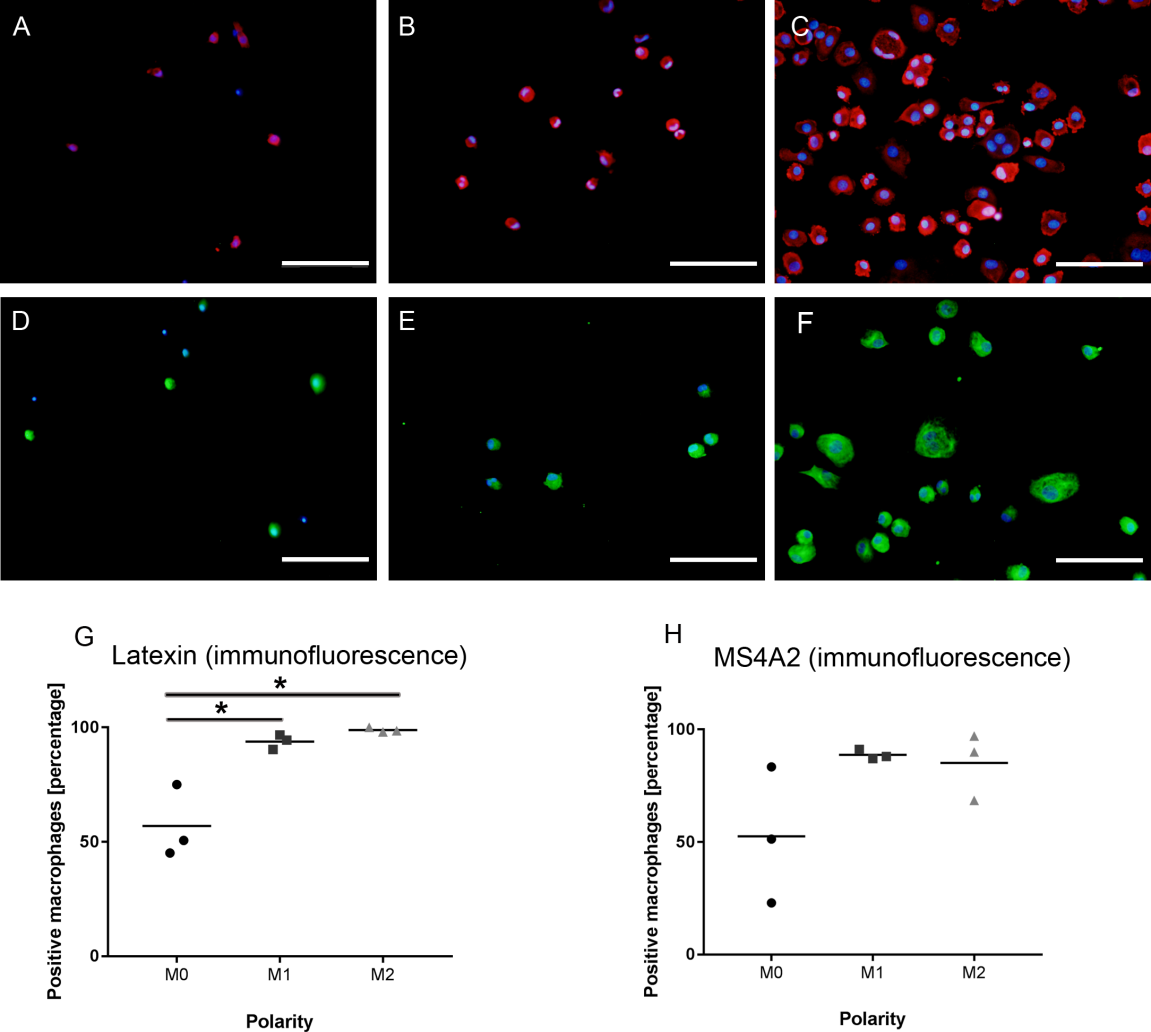
Protein expression of the predicted biomarkers latexin and MS4A2 in canine M0-, M1-, and M2-macrophages. **A)** Expression of latexin in non-stimulated canine M0-macrophages. **B)** Expression of latexin in canine M1-macrophages. **C)** Expression of latexin in canine M2-macrophages. **D)** Expression of MS4A2 in canine M0-macrophages. **E)** Expression of MS4A2 in canine M1-macrophages. **F)** Expression of MS4A2 in canine M2-macrophages. A-F) Scale bars = 100 μm. Nuclear counterstaining with bisbenzimide. **G)** Dot plots illustrating a significantly higher percentage of immunopositive cells in canine M1- and M2-macrophages as compared to M0-macrophages (One-factorial ANOVA with group-wise t-tests, astrerisk = p<0.05). **H)** Dot plot showing lack of statistical differences in the percentage of immunopositive cells for MS4A2 in canine M0-, M1-, and M2-macrophages.

In order to test, whether the biomarkers predicted to distinguish between canine M0-, M1-, and M2-macrophages during the transcriptome investigations are also mirrored by altered protein expression, an additional experiment using blood from 3 dogs was performed. The cells were isolated and polarized as described and labeled with antibodies targeting MS4A2 and LXN (Table 1). There was protein expression in canine macrophages in vitro for both molecules (Fig 6). However, the percentage of cells labeled with an antibody against MS4A2 showed no differences between M0-, M1-, and M2-macrophages (p = 0.117; Fig 6). The percentage of positive cells for LXN was significantly higher in both M1-and M2-macrophages as compared to M0-macrophages (p = 0.02 and p = 0.01, respectively); however, there was no difference in the percentage of LXN-immunopositive cells between M1- and M2-macrophages (p = 0.06; Fig 6).

## Discussion

The current investigation is the first report upon the properties of canine polarized macrophages *in vitro* with a special emphasis on the establishment of unique distinctive gene signatures. Even though *in vitro* data on macrophage polarization doubtlessly cannot be simply extrapolated to *in vivo* situations and the paradigm of M1-/M2-polarization represents a simplified approach, which depicts only two extremes of macrophage heterogeneity [8, 48–50], this initial reductionistic *in vitro* approach will provide a basis for future investigations on the role of macrophage polarization in both healthy and diseased dogs.

Striking morphological differences were observed between canine M0-, M1-, and M2-macrophages, which are most probably attributed to direct effects of cytokine stimulation. Similar to observations in other species [51–54], M1-macrophage cultures appeared to dominantly adapt an amoeboid morphology, although a significant proportion of M0-macrophages also obtained such an amoeboid morphology. Notably, M2-cultures were rather heterogeneous including spindeloid cells and MNGs, which is in line with previous observations in human and murine bone marrow- and blood-derived M2-macrophages [51–54]. Interestingly, stimulation of macrophages with IL-4 and IL-13 together with colony stimulation factors can lead to the formation of MNGs [55–58]. The recent observation of MNG formation, exclusively in canine M2-cultures, is consistent with reports from other species [44, 57, 59].

A variety of different phenotypic markers has been proposed for the differentiation of human and murine M1- and M2-macrophages [4, 31]. Even though a multitude of antibodies targeting immune cells including macrophages is known to cross-react with canine antigens [60–63], commercially available antibodies explicitly designed for the use on canine cells and tissues are frequently not available. The prototypical literature-based M1-markers CD16, CD32, and iNOS and the M2-markers CD163 and arginase-1 were demonstrated to be inappropriate for the immunophenotypic discrimination of M0-, M1-, and M2-polarization states in canine macrophages in the present study. This may in part be explained by interspecies differences [64, 65]. For instance, following classical stimulation with pro-inflammatory cytokines, murine macrophages produce NO, whereas human macrophages nearly lack synthesis of NO in response to classically activating stimuli [66, 67]. Moreover, arginase-1 has been reported as a prototype marker for murine M2-macrophages, which is however inappropriate for the detection of human M2-macrophages [13, 44, 68, 69]. Based on the present observations, canine macrophages may thus share closer similarities with human than murine macrophages, thus underlining the role of dogs in translational research. Similar to the immunofluorescence data, and supporting the results of the immunophenotyping, expression values for all probesets annotated with the canine genes NOS2 (2 probesets) and ARG1 (3 probesets), which encode iNOS and arginase-1, respectively, also lacked differences in their expression values between M0-, M1-, and M2-macrophages (data available under accession number: E-MTAB-5458; http://www.ebi.ac.uk/arrayexpress). MHC class II is sometimes regarded as a marker for classically activated M1 macrophages [70]; however, a substantial fundus of literature also indicates MHC class II as a pan-macrophage marker, which is expressed on both M1 and M2 macrophages in mice and humans without discriminating between both phenotypes [4, 7, 68]. Concordantly, the immunofluorescence data demonstrated that MHC class II was found to distinguish between canine M2- and M0-macrophages with a lower expression in the latter (Fig 3). In fact, it is well known that IL-4 acts as a potent activator of macrophages and induces an up-regulation of MHC class II [23, 71–75]. However, it must be critically considered that MHC class II appeared inappropriate for the differentiation of canine M0- and M1-macrophages on the protein level, which contradicts reported up-regulation of MHC class II on M1 macrophages [7]. Whether these differences are attributed to true species effects or whether the low number of investigated animals and the quantification method (immunofluorescence) are responsible for not reaching the level of significance remains to be determined.

Conclusively, it is unlikely that a single antibody will be sufficient to specifically detect canine M1- and M2-macrophages *in vivo*. Due to the paucity of commercially available antibodies detecting canine antigens this will probably also involve the generation of antibodies targeting canine epitopes.

The present study thus aimed to set up a transcriptomic basis that should encourage future attempts to establish a broader antibody panel against antigens, which are predicted to potentially represent discriminating markers for canine macrophage phenotypes. Interestingly, in the present study, CD206 was among the top regulated genes of canine M2 macrophages on the transcriptome level, and the results of the immunophenotyping also validated CD206 as a promising marker for canine M2 polarized macrophages. This implies that CD206 might constitute a conserved marker, which is appropriate for labeling M2-macrophages in various species including the dog [4, 27, 31, 72, 76]. However, a limitation of the present study is that the suitability of markers identified in the transcriptome analyses either still needs to be verified on the protein level or produced partially conflicting results in the immunofluorescence investigations (*i*.*e*. CD163, LXN, and MS4A2).

For M1-associated up-regulated genes, multiple metabolic pathways such as “steroid biosynthesis”, and “glycolysis/gluconeogenesis” demonstrated to be enriched in the functional annotation of microarray data. Human and murine monocyte to macrophage differentiation has previously been shown to go along with profound changes in the lipid metabolism as a prerequisite for phagocytosis [77]. Moreover, in the hierarchical clustering analysis, the M1-specific cluster was functionally annotated to biological processes involved in “respiratory burst”. M1 macrophages produce a variety of pro-inflammatory mediators including ROS, whereas in contrast, IL-4 inhibits the respiratory burst [78].

Enriched terms related to the peroxisome proliferator activated receptor (PPAR) signaling pathway were retrieved for the comparison of M2 *vs*. M1 up-regulated genes. PPARγ is a member of the nuclear receptor superfamily with potent anti-inflammatory properties and regulatory functions in fatty acid metabolism [79–82]. Interestingly, PPARγ agonists such as rosiglitazone induce an alternative M2a-activation state in murine macrophages and have been used as neuroprotective agents [83, 84]. Pharmacological approaches, designed to enhance M2-dominated immune responses may thus similarly represent a promising tool in canine diseases.

Interestingly, multiple biological processes related to an enhanced cell cycle were enriched in canine M2-macrophages as compared to both M0- and M1-macrophages. IL-4 has previously been reported to induce local macrophage proliferation in the context of chronic inflammation [59]. Moreover, human monocyte to macrophage differentiation in the presence of M-CSF is associated with a dramatic regulation of multiple cell-cycle genes [44]. The transcriptomic link to enhanced cell cycle and proliferation is probably also reflected by the higher cell number of M2-macrophages in the present study as compared to M0-macrophages (S1 Fig).

Comparison of the canine transcriptome data with published murine and human prototype markers demonstrated a relatively low overlap. This confirms recent reports on marked interspecies differences between polarized macrophages [13] and suggests that some properties of polarized macrophages are unique to the dog, demonstrating that reported literature-based markers cannot simply be transferred to another species.

Based on this observation we sought to predict novel transcriptomic markers using a hypothesis-driven approach. Using a correlation-based algorithm, the carboxypeptidase inhibitor *latexin* (*LXN*) [85–88] was retrieved for canine M1-macrophages. Though *LXN*-expression by murine macrophages upon pro-inflammatory stimulation has been demonstrated [85], *LXN* has so far not been proposed as a marker for M1-macrophages in the literature for any species. For the canine M2-phenotype, the high-affinity receptor for IgE *membrane-spanning 4-domains*, *subfamily A*, *member 2* (*MS4A2*) was predicted to represent the most powerful biomarker. Interestingly, other members of the molecule family, *i*.*e*. *MS4A4A* and *MS4A6A*, have been previously shown to be associated with M2-polarization [4, 44, 89]. In an attempt to validate *LXN* and *MS4A2* as markers for canine macrophages, we demonstrated that MS4A2 showed no differences in the percentage of immunopositive cells between the three conditions. The percentage of immunopositive cells for LXN was higher in both M1- and M2-macrophages as compared to M0-macrophages. However, similar to MS4A2, LXN failed to distinguish between canine M1- and M2-macrophages. Thus, similar to CD163, the protein data did not accurately mirror the transcriptomic prediction. This could be attributed to conflicting differences between RNA- and protein level for these molecules. However, the low number of dogs tested and the fact that low level differences in protein expression may not be detected by immunofluorescence have certainly influenced the statistical power and sensitivity, and thus future validating experiments both in canine tissues and *in vitro* remain to be performed.

## Supporting information

S1 Fig Dot plot diagrams depicting differences in the absolute number of cells per field (200x) in canine M0-, M1-, and M2-macrophages derived from 3 dogs on day 7 in culture. One-factorial ANOVA with group-wise t tests reveals a significantly higher number of cells in M2-polarized macrophages as compared to non-stimulated (M0)-macrophages (p≤0.05; asterisk).
(TIF)

S1 Table List of the genes included in the M1-associated cluster of the hierarchical clustering analysis (refer to Fig 3). (DOCX)

S2 Table List of the genes included in the M2-associated cluster of the hierarchical clustering analysis (refer to Fig 3). (DOCX)

S3 Table Overview on retrieved enriched gene ontology biological processes and KEGG pathways of the clusters, resulting from the hierarchical clustering analysis (refer to Fig 3). (DOCX)

S4 Table List for the intersections of differentially expressed genes in the comparison of literature-based human and murine markers with canine M1- and M2-associated genes as retrieved by the present study (refer to Fig 4). Excel table.
(XLSX)

S5 Table Lists of differentially expressed M1- and M2-macrophage associated probesets with fold change. Sheet 1 depicts all genes, which were upregulated in M1- vs. M0-macrophages and simultaneously downregulated in M2- vs M1-macrophages (i.e. canine M1-macrophage genes). Sheet 2 shows all genes, which were upregulated in M2- vs. M0-macrophages and simultaneously upregulated in M2- vs M1-macrophages (i.e. canine M2-macrophage genes).
(XLSX)

S6 Table Selected biomarkers predicted to discriminate between canine M1- and M2- macrophages as retrieved and ranked by Prophet. (DOCX)
