# Supplementary figures and images for: Morphologic, phenotypic, and transcriptomic characterization of classically and alternatively activated canine blood-derived macrophages in vitro
Source: PLoS One. 2017 Aug 17;12(8):e0183572. doi: 10.1371/journal.pone.0183572 (PMC5560737; doi:10.1371/journal.pone.0183572)

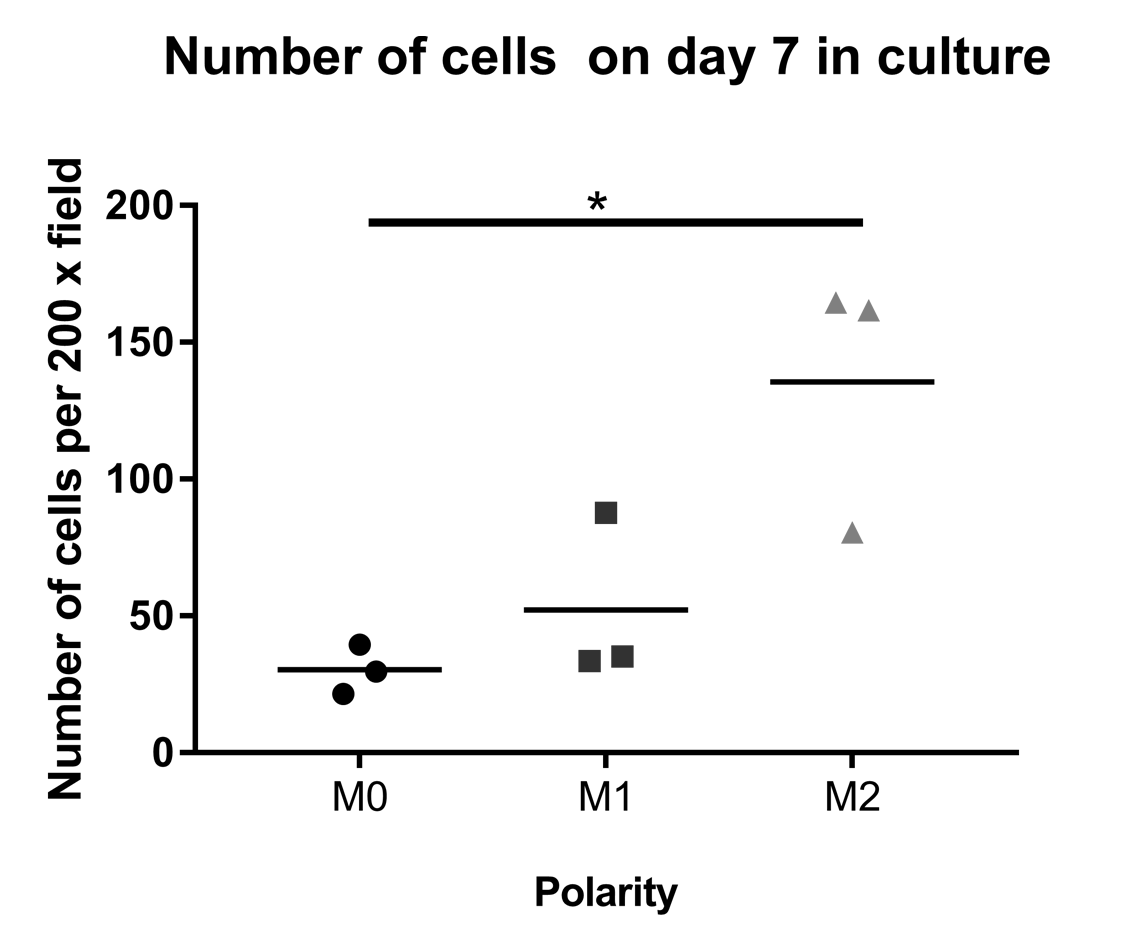

Supplement: S1 Fig — One-factorial ANOVA with group-wise t tests reveals a significantly higher number of cells in M2-polarized macrophages as compared to non-stimulated (M0)-macrophages (p≤0.05; asterisk). (TIF) [file pone.0183572.s001.tif]
