# Supplementary material for: Morphologic, phenotypic, and transcriptomic characterization of classically and alternatively activated canine blood-derived macrophages in vitro
Source: PLoS One. 2017 Aug 17;12(8):e0183572. doi: 10.1371/journal.pone.0183572 (PMC5560737; doi:10.1371/journal.pone.0183572)
Supplement: S1 Table — (DOCX) [file pone.0183572.s002.docx]

**S1 Table: List of the genes included in the M1-associated cluster of the hierarchical clustering analysis (refer to figure 3).**

| **Gene name** | **Gene symbol** | **Fold change**  **(M1 *vs.* M0)** |
| --- | --- | --- |
| Interferon, alpha-inducible protein 6 | IFI6 | 269.13 |
| Epoxide hydrolase 2, cytoplasmic | EPHX2 | 119.72 |
| E3 ubiquitin-protein ligase NEURL3-like | LOC102152163 | 86.2 |
| Nuclear protein, transcriptional regulator, 1 | NUPR1 | 80.16 |
| Chemokine (C-C motif) ligand 20 | CCL20 | 67.9 |
| Syndecan 4 | SDC4 | 58.61 |
| WNK lysine deficient protein kinase 2 | WNK2 | 52.35 |
| Tumor necrosis factor | TNF | 47.2 |
| Chromosome 18 open reading frame, human C7orf10 | C18H7orf10 | 43.19 |
| Bone morphogenetic protein 1 | BMP1 | 43.14 |
| Fascin homolog 1, actin-bundling protein (Strongylocentrotus purpuratus) | FSCN1 | 35.83 |
| Nucleoredoxin | NXN | 34.07 |
| Chemokine (C-X-C motif) receptor 3 | CXCR3 | 32.55 |
| MOB kinase activator 3B | MOB3B | 29.21 |
| Indoleamine 2,3-dioxygenase 2 | IDO2 | 28.65 |
| Inhibin, beta A | INHBA | 27.8 |
| UDP-N-acetyl-alpha-D-galactosamine:polypeptide N-acetylgalactosaminyltransferase 3 (GalNAc-T3) | GALNT3 | 23.07 |
| Hook microtubule-tethering protein 1 | HOOK1 | 23.06 |
| Glutaminyl-peptide cyclotransferase | QPCT | 22.78 |
| Kell blood group glycoprotein-like | LOC100855519 | 22.58 |
| Transmembrane protein 176A | TMEM176A | 19.58 |
| Maltase-glucoamylase (alpha-glucosidase) | MGAM | 18.37 |
| Peptidase inhibitor 3, skin-derived | PI3 | 17.25 |
| Dmx-like 2 | DMXL2 | 17.25 |
| Placenta-specific gene 8 protein-like | LOC608687 | 16.4 |
| Lysine (K)-specific demethylase 4D | KDM4D | 16.12 |
| Solute carrier family 22, member 15 | SLC22A15 | 15.67 |
| Acyl-coenzyme A amino acid N-acyltransferase 2-like | LOC481634 | 15.65 |
| Hyaluronan synthase 2 | HAS2 | 14.43 |
| Hydroxycarboxylic acid receptor 3 | HCAR3 | 13.88 |
| Interleukin 17F | IL17F | 13.57 |
| Solute carrier family 39 (zinc transporter), member 14 | SLC39A14 | 13.48 |
| Dual specificity phosphatase 10 | DUSP10 | 12.98 |
| TRAF3 interacting protein 2 | TRAF3IP2 | 12.94 |
| Complement component 1, s subcomponent | C1S | 12.93 |
| Tetraspanin 9 | TSPAN9 | 12.18 |
| Monoglyceride lipase | MGLL | 12.07 |
| Free fatty acid receptor 2 | FFAR2 | 11.51 |
| Interleukin-1 receptor-associated kinase 3 | IRAK3 | 10.7 |
| Von Willebrand factor A domain containing 5A | VWA5A | 10.24 |
| Glycosyltransferase 1 domain containing 1 | GLT1D1 | 9.45 |
| STEAP family member 4 | STEAP4 | 8.28 |
| Serpin peptidase inhibitor, clade G (C1 inhibitor), member 1 | SERPING1 | 7.12 |
| Multiple C2 domains, transmembrane 1 | MCTP1 | 7.0 |
| Multiple C2 domains, transmembrane 2 | MCTP2 | 6.25 |
| Xanthine dehydrogenase | XDH | 5.8 |
| Cochlin | COCH | 5.22 |
| DENN/MADD domain containing 5A | DENND5A | 5.11 |
| Neutrophil cytosolic factor 1 | NCF1 | 4.95 |
| Adenosylhomocysteinase-like 2 | AHCYL2 | 3.73 |
| Kelch repeat and BTB (POZ) domain containing 7 | KBTBD7 | 3.64 |
| RNA binding protein with multiple splicing | RBPMS | 3.47 |
| Mucolipin 2 | MCOLN2 | 3.22 |
| Baculoviral IAP repeat containing 3 | BIRC3 | 3.05 |
| Proline rich 5 like | PRR5L | 2.96 |
| Spindlin family, member 2B | SPIN2B | 2.7 |
| Mesoderm induction early response 1, family member 3 | MIER3 | 2.54 |
| SIK family kinase 3 | SIK3 | 2.39 |
| Acyl-CoA synthetase long-chain family member 1 | ACSL1 | 2.37 |
| 1-acylglycerol-3-phosphate O-acyltransferase ABHD5-like | LOC485570 | 2.26 |
| Oxysterol binding protein-like 6 | OSBPL6 | 2.2 |
| M-phase phosphoprotein 6 | MPHOSPH6 | 2.11 |
| Ras association (RalGDS/AF-6) domain family member 4 | RASSF4 | 2.04 |
| EH-domain containing 1 | EHD1 | 1.84 |
| DCN1, defective in cullin neddylation 1, domain containing 1 | DCUN1D1 | 1.78 |
| Protein kinase, X-linked | PRKX | 1.77 |
| Chromosome 11 open reading frame, human C9orf72 | C11H9orf72 | 1.72 |
| NGFI-A binding protein 1 (EGR1 binding protein 1) | NAB1 | 1.7 |
| Family with sequence similarity 149, member A | FAM149A | 1.67 |
| LYR motif containing 1 | LYRM1 | 1.67 |
| RAB GTPase activating protein 1-like | RABGAP1L | 1.61 |
| Tumor necrosis factor (ligand) superfamily, member 13b | TNFSF13B | 1.58 |
| Chromosome 12 open reading frame, human C6orf106 | C12H6orf106 | 1.55 |
| Endoplasmic reticulum aminopeptidase 2 | ERAP2 | 1.33 |
| Signal transducer and activator of transcription 1-like | LOC488449 | 1.32 |
| Apolipoprotein L3-like | LOC102156332 | 1.21 |
| KIAA1430 ortholog | KIAA1430 | 1.11 |
| NEDD4 binding protein 1 | N4BP1 | 1.05 |
| AlkB, alkylation repair homolog 1 (E. coli) | ALKBH1 | -1.06 |
| Polyhomeotic homolog 3 (Drosophila) | PHC3 | -1.1 |
| Chromosome 6 open reading frame, human C16orf52 | C6H16orf52 | -1.42 |
