# Supplementary material for: Morphologic, phenotypic, and transcriptomic characterization of classically and alternatively activated canine blood-derived macrophages in vitro
Source: PLoS One. 2017 Aug 17;12(8):e0183572. doi: 10.1371/journal.pone.0183572 (PMC5560737; doi:10.1371/journal.pone.0183572)
Supplement: S2 Table — (DOCX) [file pone.0183572.s003.docx]

**S2 Table: List of the genes included in the M2-associated cluster of the hierarchical clustering analysis (refer to figure 3).**

| **Gene name** | **Gene symbol** | **Fold change**  **(M2 *vs.* M0)** |
| --- | --- | --- |
| Sodium channel, voltage-gated, type II, beta subunit | SCN2B | 1061.28 |
| Chemokine (C-C motif) ligand 24 | CCL24 | 1060.82 |
| Nephronectin | NPNT | 444.07 |
| Lymphatic vessel endothelial hyaluronan receptor 1 | LYVE1 | 322.32 |
| Caldesmon 1 | CALD1 | 224.66 |
| Guanine deaminase | GDA | 174.44 |
| Interleukin 13 receptor, alpha 2 | IL13RA2 | 170.59 |
| Fructose-1,6-bisphosphatase 1 | FBP1 | 160.02 |
| Ubiquitin-conjugating enzyme E2 C-like | LOC481325 | 151.23 |
| SHC SH2-domain binding protein 1 | SHCBP1 | 117.77 |
| Dynamin 1 | DNM1 | 112.78 |
| Junctional adhesion molecule 3 | JAM3 | 78.46 |
| Kinesin family member 23 | KIF23 | 66.78 |
| Kinesin family member 11 | KIF11 | 64.2 |
| Cfa-mir-125b-2 | cfa-mir-125b-2 | 58.78 |
| NUF2, NDC80 kinetochore complex component | NUF2 | 54.62 |
| C-type lectin domain family 4, member G | CLEC4G | 54.14 |
| Cyclin B2 | CCNB2 | 48.95 |
| Cyclin B1 | CCNB1 | 46.92 |
| Complement component 3 | C3 | 44.14 |
| Discs, large (Drosophila) homolog-associated protein 5 | DLGAP5 | 39.12 |
| Epithelial cell transforming sequence 2 oncogene | ECT2 | 34.87 |
| Meiotic nuclear divisions 1 homolog (S. cerevisiae) | MND1 | 32.93 |
| MORN repeat containing 2 | MORN2 | 32.83 |
| High mobility group box 3 | HMGB3 | 32.52 |
| Fms-related tyrosine kinase 1 (vascular endothelial growth factor/vascular permeability factor receptor) | FLT1 | 28.92 |
| Antigen KI-67-like | LOC102152056 | 28.65 |
| Pituitary tumor-transforming 1 | PTTG1 | 26.01 |
| Actinin, alpha 1 | ACTN1 | 25.88 |
| mitotic spindle assembly checkpoint protein MAD2A-like | LOC476070 | 22.41 |
| Tectonic family member 2 | TCTN2 | 20.56 |
| Myosin binding protein C, fast type | MYBPC2 | 20.25 |
| Myosin IE | MYO1E | 18.86 |
| Origin recognition complex, subunit 6 | ORC6 | 14.6 |
| Myosin, light chain 4, alkali; atrial, embryonic | MYL4 | 14.4 |
| Alanyl (membrane) aminopeptidase | ANPEP | 14.09 |
| Adenylate cyclase 4 | ADCY4 | 14.07 |
| Biphenyl hydrolase-like (serine hydrolase) | BPHL | 13.73 |
| Schwannomin interacting protein 1 | SCHIP1 | 13.19 |
| High mobility group box 3 | HMGB3 | 13.05 |
| Chromosome 27 open reading frame, human C12orf5 | C27H12orf5 | 11.72 |
| Crystallin, lambda 1 | CRYL1 | 10.33 |
| Coiled-coil domain containing 80 | CCDC80 | 9.47 |
| Ribosomal RNA processing 8, methyltransferase, homolog (yeast) | RRP8 | 9.43 |
| Mannosidase, alpha, class 1C, member 1 | MAN1C1 | 9.41 |
| Centromere protein H | CENPH | 8.48 |
| Non-SMC condensin I complex, subunit H | NCAPH | 8.36 |
| Phosphoenolpyruvate carboxykinase 2 (mitochondrial) | PCK2 | 8.3 |
| X-prolyl aminopeptidase (aminopeptidase P) 1, soluble | XPNPEP1 | 8.23 |
| Nucleolar and spindle associated protein 1 | NUSAP1 | 8.2 |
| Solute carrier family 26 (anion exchanger), member 2 | SLC26A2 | 7.8 |
| RAD9-HUS1-RAD1 interacting nuclear orphan 1 | RHNO1 | 7.8 |
| SPC25, NDC80 kinetochore complex component | SPC25 | 7.67 |
| WD repeat domain 11 | WDR11 | 7.5 |
| Breast cancer anti-estrogen resistance 3 | BCAR3 | 7.23 |
| G-protein signaling modulator 2 | GPSM2 | 7.21 |
| Asparagine synthetase [glutamine-hydrolyzing]-like | LOC475240 | 7.11 |
| E2F transcription factor 2 | E2F2 | 7 |
| Sestrin 3 | SESN3 | 6.91 |
| Cyclin E2 | CCNE2 | 6.65 |
| Cyclin-dependent kinase 2 associated protein 1 | CDK2AP1 | 6.34 |
| ryanodine receptor 1 (skeletal) | RYR1 | 6.14 |
| Aurora kinase B | AURKB | 6.01 |
| Transmembrane 6 superfamily member 1 | TM6SF1 | 5.97 |
| EGF-like module-containing mucin-like hormone receptor-like 4-like | LOC100855754 | 5.96 |
| Fanconi anemia, complementation group D2 | FANCD2 | 5.89 |
| Cyclin-dependent kinase 1 | CDK1 | 5.68 |
| Family with sequence similarity 136, member A | FAM136A | 5.6 |
| Cms1 ribosomal small subunit homolog (yeast) | CMSS1 | 4.69 |
| Aurora kinase A | AURKA | 4.51 |
| SET domain containing (lysine methyltransferase) 7 | SETD7 | 4.45 |
| NDC80 kinetochore complex component | NDC80 | 4.39 |
| Ubiquitin protein ligase E3D | UBE3D | 3.84 |
| Centromere protein P | CENPP | 3.71 |
| Transcription factor B1, mitochondrial | TFB1M | 3.49 |
| Reticulon 4 interacting protein 1 | RTN4IP1 | 3.29 |
| Cell division cycle associated 7-like | CDCA7L | 3.26 |
| Leucine-rich pentatricopeptide repeat containing | LRPPRC | 3.22 |
| Polymerase (RNA) I polypeptide E, 53kDa | POLR1E | 3.22 |
| Prolyl-tRNA synthetase 2, mitochondrial (putative) | PARS2 | 3.21 |
| GTP-binding protein 8 (putative) | GTPBP8 | 3.17 |
| Methylenetetrahydrofolate dehydrogenase (NADP+ dependent) 1-like | MTHFD1L | 3.15 |
| TNF receptor-associated protein 1 | TRAP1 | 3.08 |
| Potassium voltage-gated channel, shaker-related subfamily, beta member 2 | KCNAB2 | 3.06 |
| Mitochondrial ribosomal protein S10 | MRPS10 | 2.79 |
| Acylaminoacyl-peptide hydrolase | APEH | 2.79 |
| Chromobox homolog 5 | CBX5 | 2.67 |
| Ribonuclease P/MRP 30kDa subunit | RPP30 | 2.51 |
| Carnitine O-octanoyltransferase | CROT | 2.5 |
| Dynamin-like 120 kDa protein, mitochondrial-like | LOC477129 | 2.49 |
| Golgi integral membrane protein 4 | GOLIM4 | 2.31 |
| Mitochondrial calcium uniporter regulator 1 | MCUR1 | 2.3 |
| PIN2/TERF1 interacting, telomerase inhibitor 1 | PINX1 | 2.29 |
| Cytoskeleton associated protein 2 | CKAP2 | 2.27 |
| Translocation associated membrane protein 2 | TRAM2 | 2.26 |
| Nucleophosmin/nucleoplasmin 3 | NPM3 | 2.26 |
| Solute carrier family 1 (neutral amino acid transporter), member 5 | SLC1A5 | 2.21 |
| Solute carrier family 39 (zinc transporter), member 6 | SLC39A6 | 2.14 |
| Nicotinamide nucleotide transhydrogenase | NNT | 2.13 |
| Mannosidase, alpha, class 1A, member 1 | MAN1A1 | 2.12 |
| Mediator complex subunit 28 | MED28 | 2.1 |
| N(alpha)-acetyltransferase 20, NatB catalytic subunit | NAA20 | 2.01 |
| Peptidyl-prolyl cis-trans isomerase NIMA-interacting 4-like | LOC102151798 | 1.88 |
| Pentatricopeptide repeat domain 3 | PTCD3 | 1.51 |
| G patch domain containing 4 | GPATCH4 | 1.42 |
