# Supplementary material for: Morphologic, phenotypic, and transcriptomic characterization of classically and alternatively activated canine blood-derived macrophages in vitro
Source: PLoS One. 2017 Aug 17;12(8):e0183572. doi: 10.1371/journal.pone.0183572 (PMC5560737; doi:10.1371/journal.pone.0183572)
Supplement: S3 Table — (DOCX) [file pone.0183572.s004.docx]

**S3 Table: Overview on retrieved enriched gene ontology biological processes and KEGG pathways of the clusters, resulting from the hierarchical clustering analysis (refer to figure 3).**

| **Hierarchical cluster** | **Enriched biological process categories*** | **Enriched KEGG pathways*** |
| --- | --- | --- |
| **I** | - Peptidyl-lysine mono- and dimethylation | - n.s. |
| **II** | - n.s. | - Glycosaminoglycan biosynthesis |
| **III** | - Immune response-activating signal transduction | - T and B cell receptor signaling pathway - Toll-like receptor signaling pathway |
| **IV** | - Monosaccharide metabolic process - Organic substance catabolic process - Cellular catabolic process | - Metabolic pathways |
| **V** | - Response to other organism - Regulation of lymphocyte proliferation | - Metabolic pathways |
| **VI** | - M phase of mitotic cell cycle - Mitotic spindle organization | - Oocyte meiosis - Cell cycle |
| **VII** | - tRNA aminoacylation for protein translation | - Metabolic pathways |
| **VIII** | - Respiratory burst involved in defense response | - Chemokine signaling pathway - Apoptosis - Peroxisome |
| **IX** | - Cytokinesis - Antigen receptor-mediated signaling pathway | - n.s. |

*employing Web-based Gene Set Analysis Toolkit (WebGestalt; http://bioinfo.vanderbilt.edu/webgestalt/) with default settings; adjusted p-value ≤0.05;

n.s., no significantly enriched biological process categories
