# Supplementary material for: Morphologic, phenotypic, and transcriptomic characterization of classically and alternatively activated canine blood-derived macrophages in vitro
Source: PLoS One. 2017 Aug 17;12(8):e0183572. doi: 10.1371/journal.pone.0183572 (PMC5560737; doi:10.1371/journal.pone.0183572)
Supplement: S6 Table — (DOCX) [file pone.0183572.s007.docx]

**S5 Table: Selected biomarkers predicted to discriminate between canine M1- and M2- macrophages as retrieved and ranked by Prophet.**

| **ProbeSetID** | **Gene symbol**  **(canine)** | **M1 *vs.* M0** | | **M2 *vs.* M0** | | **M2 *vs.* M1** | | |
| --- | --- | --- | --- | --- | --- | --- | --- | --- |
|  |  | **Fold change** | **q-value** | **Fold change** | **q-value** | **Fold change** | **q-value** | |
| *Predicted M1-markers* | | | | | | | | |
| CfaAffx.14358.1.S1_at | LXN | 4.68 | 0.0000 | -1.68 | 0.0000 | -7.88 | | 0.0000 |
| Cfa.5195.1.A1_s_at | LXN | 4.02 | 0.0000 | -1.57 | 0.0002 | -6.33 | | 0.0000 |
| Cfa.5195.1.A1_at | LXN | 3.33 | 0.0000 | -1.64 | 0.0001 | -5.46 | | 0.0000 |
| Cfa.14007.1.A1_x_at | LXN | 3.72 | 0.0000 | -1.63 | 0.0011 | -6.06 | | 0.0000 |
| Cfa.8008.1.A1_at | --- | 1.70 | 0.0000 | -1.26 | 0.0050 | -2.14 | | 0.0000 |
| Cfa.21216.1.S1_s_at | ST3GAL5 | 4.84 | 0.0000 | -1.89 | 0.0024 | -9.15 | | 0.0000 |
| Cfa.15365.1.A1_at | ST3GAL5 | 3.80 | 0.0000 | -1.79 | 0.0028 | -6.81 | | 0.0000 |
| CfaAffx.1855.1.S1_s_at | ZDHHC14 | 14.21 | 0.0000 | 2.18 | 0.0027 | -6.51 | | 0.0000 |
| Cfa.1692.1.A1_at | KIF1B | 2.64 | 0.0000 | 1.44 | 0.0001 | -1.84 | | 0.0000 |
| Cfa.10411.1.A1_at | LPGAT1 | 2.61 | 0.0000 | -1.16 | 0.2604 | -3.01 | | 0.0000 |
| *Predicted M2-markers* | | | | | | | | |
| Cfa.3662.1.S1_at | MS4A2 | -2.51 | 0.0227 | 54.98 | 0.0000 | 137.95 | 0.0000 | |
| Cfa.12095.1.A1_at | INTS4 | -1.30 | 0.0157 | 2.30 | 0.0000 | 3.00 | 0.0000 | |
| Cfa.11745.1.A1_at | EEF1E1 | 1.46 | 0.0146 | 4.42 | 0.0000 | 3.03 | 0.0000 | |
| Cfa.11769.1.A1_at | MORN2 | 3.39 | 0.0010 | 32.83 | 0.0000 | 9.67 | 0.0000 | |
| Cfa.10515.1.S1_at | WDR12 | 1.40 | 0.0084 | 2.81 | 0.0000 | 2.01 | 0.0000 | |
| CfaAffx.22128.1.S1_at | S100P | 1.75 | 0.0700 | 8.37 | 0.0000 | 4.78 | 0.0002 | |
| Cfa.15054.1.A1_s_at | DST | 3.35 | 0.0020 | 15.12 | 0.0000 | 4.51 | 0.0005 | |
| Cfa.19503.1.S1_at | EXTL2 | 1.59 | 0.0002 | 2.94 | 0.0000 | 1.84 | 0.0000 | |
| CfaAffx.13326.1.S1_at | DPH6 | 1.37 | 0.0020 | 2.20 | 0.0000 | 1.60 | 0.0001 | |
| Cfa.1725.1.S1_at | TMEM67 | 3.75 | 0.0003 | 17.30 | 0.0000 | 4.61 | 0.0001 | |
